# A counseling program on nuisance bleeding improves quality of life in patients on dual antiplatelet therapy: A randomized controlled trial

**DOI:** 10.1371/journal.pone.0182124

**Published:** 2017-08-23

**Authors:** Simone Biscaglia, Elisabetta Tonet, Rita Pavasini, Matteo Serenelli, Giulia Bugani, Paolo Cimaglia, Francesco Gallo, Giosafat Spitaleri, Annamaria Del Franco, Giorgio Aquila, Francesco Vieceli Dalla Sega, Matteo Tebaldi, Carlo Tumscitz, Roberto Ferrari, Gianluca Campo

**Affiliations:** 1 Cardiovascular Section, Medical Sciences Department, Azienda Ospedaliera Universitaria S.Anna, Ferrara, Italy; 2 Department of Morphology, Surgery and Experimental Medicine, University of Ferrara, Ferrara, Italy; 3 Department of Medical Sciences, University of Ferrara, Ferrara, Italy; 4 University of Ferrara, Ferrara, Italy; Maria Cecilia Hospital, GVM Care&Research, E.S: Health Science Foundation, Cotignola, Italy; 5 Laboratory for Technologies of Advanced Therapies (LTTA), University of Ferrara, Ferrara, Italy; Kurume University School of Medicine, JAPAN

## Abstract

**Background:**

Nuisance bleeding is a major determinant of quality of life and drug discontinuation in patients on dual antiplatelet therapy (DAPT). However, no randomized trial has been focused on the impact of nuisance bleeding on quality of life.

**Methods:**

BATMAN is an investigator-driven, randomized, controlled, single-center, open trial (NCT02554006). Four hundred and forty-eight consecutive patients with indication to at least 6 months of DAPT were randomized to: i) multimodal counseling program focused on nuisance bleedings (interventional arm); ii) usual discharge process (control arm). The primary endpoint was the one-month health-related quality of life assessed by the EuroQol-5 Dimension (EQ-5D) visual analog scale (VAS) score. Secondary endpoints were EQ-5D at 1 and 6 months, EQ-5D VAS at 6 months, DAPT withdrawal, need of information regarding DAPT and/or nuisance bleedings, 6-month ischemic and bleeding adverse events.

**Results:**

The EQ5D-VAS was significantly higher in the interventional arm compared to the control arm at 1 and 6 months (81[74–88] vs. 73[64–80], p < 0.001 at 1 month; 82[76–88] vs. 74[65–81], p < 0.001 at 6 months). Patients in the interventional arm had also significantly lower pain/discomfort and anxiety/depression at the EQ-5D both at 1 and 6 months. Patients in the control arm withdrew DAPT significantly more (7 (3%) vs. 1 (0.4%), p = 0.03) and looked for information regarding DAPT and/or about nuisance bleeding more frequently than those in the interventional arm (178 (79%) vs.19 (8%), p < 0.001).

**Conclusions:**

The systematic utilization of a multimodal counseling program improved quality of life and reduced the DAPT withdrawal rate in patients on DAPT.

## Introduction

Nuisance bleeding is a major determinant of dual antiplatelet therapy (DAPT) discontinuation, often leading to adverse events and re-hospitalizations, in patients post-percutaneous coronary intervention (PCI) [1–6]. The wide-spread use of newer and more potent P2Y12 inhibitors and the prolongation of DAPT length in some settings have further heightened incidence and impact of nuisance bleedings [4, 7–9]. All published studies share a high percentage of patients experiencing nuisance bleedings during DAPT. Alexopoulos et al reported a 35% rate of nuisance (Bleeding Academic Research Consortium (BARC) 1) bleedings at one month in patients treated with ticagrelor [5]. Nuisance bleedings, besides their high incidence, frequently cause DAPT withdrawal as showed by the 15% rate of prasugrel discontinuation in patients with nuisance bleeding showed by Armero et al [6] and by the 30% rate of ticagrelor discontinuation during the follow-up (33 months) reported by Bonaca et al [9] in an analysis from Ticagrelor Compared to Placebo on a Background of Aspirin–Thrombolysis in Myocardial Infarction 54 (PEGASUS-TIMI 54) trial, mostly because of nuisance bleedings. Moreover, nuisance bleedings cause also a significant reduction in quality of life and utility in patients on DAPT [3, 10]. Given all these premises, it is surprising that no randomized trial has ever been focused on nuisance bleedings. At the same time, in everyday clinical practice, nuisance bleedings represent a frequent and relevant issue and strategies able to reduce their impact are clearly on demand. Consequently, we designed “The predischarge Bundle for pATients in dual antiplatelet therapy to Minimize the negAtive impact of Nuisance bleedings on quality of life: a randomized controlled (BATMAN) trial” to test whether a counseling program minimizes the negative impact of nuisance bleeding on quality of life of patients on DAPT.

## Materials and methods

BATMAN is an investigator-driven, randomized controlled, single-center, open trial. The trial was approved by the Ethics Committee of the Ferrara University Hospital and registered on clinicaltrials.gov (NCT02554006). Informed consent was obtained from each patient and the study protocol conforms to the ethical guidelines of the 1975 Declaration of Helsinki. Major inclusion criteria were: hospital admission for ischemic heart disease with clinical indication to coronary artery angiography; successful PCI with second generation drug eluting stent (DES) implantation; indication to at least 6 months of DAPT. Major exclusion criteria were: known allergy to acetylsalicylic acid or clopidogrel or ticagrelor or prasugrel; history of bleeding diathesis; prior, present or foreseeable indication to oral anticoagulant therapy; bleeding event in the 30 days before the enrollment; pregnancy; life expectancy <12 months; participation in another trial; planned cardiac or non-cardiac surgery; patients’ inability to come to the follow-up visit (logistical reasons, patient’s unwillingness); inability to provide informed consent.

### Study population

**Fig 1:**
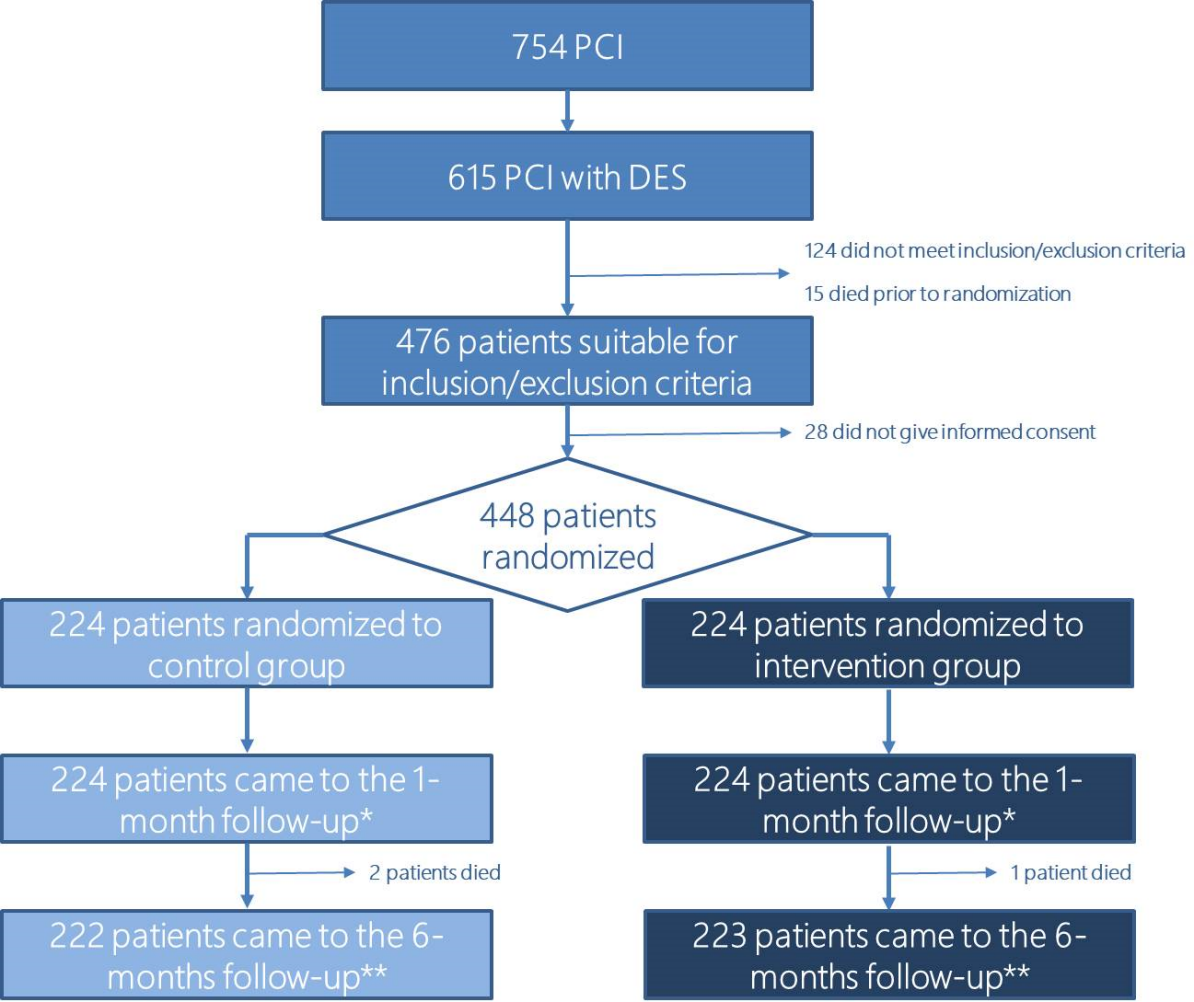
CONSORT study flow chart. * were included in the primary endpoint analysis. ** were included in the secondary endpoints 6-month analysis. PCI: percutaneous coronary intervention. DES: drug eluting stent.

From September 2015 to March 2016, 448 consecutive patients fulfilled the inclusion/exclusion criteria, gave written informed consent, and were randomized to either interventional or control group (see Fig 1 for detailed study flow chart). Randomization was performed by an independent study coordinator via sealed envelopes. To minimize potential confounding effect, randomization was stratified according to age (<70 years vs. ≥70 years), gender (male vs. female), presence of renal failure (CrCl <45 ml/min vs. ≥45 ml/min) and P2Y12 inhibitor (clopidogrel vs. ticagrelor/prasugrel). Antiplatelet agents choice was left to the physician following international guidelines [11,12]. Patients’ allocation was not masked.

### Counseling program (interventional arm)

Patients randomized to the interventional arm received (see S1 File for detailed description regarding study intervention):

1. a 15-minutes meeting with a member of the research team 24 hours prior discharge. During this visit, a core set of DAPT risks were addressed with the help of a PowerPoint presentation (see S2 File). DAPT advantages and side effects were described. The importance of compliance and the correct management of side effects (especially BARC 1 bleedings) were discussed;
2. a 15-minutes meeting with a member of the research team with a patient’s next of kin addressing the same issues: DAPT advantages and side effects, importance of compliance, side effects management;
3. a brochure describing DAPT advantages, side effects, and management (see S3 File);
4. a brochure for the patient’s general practitioner aimed at presenting DAPT rationale and management (see S4 File). At the same time, study investigators directly contacted the patient’s general practitioner by phone and/or by email describing and explaining the same topics and the clinical picture of his/her patient (see S1 File);
5. a phone number to further discuss potential side effects of DAPT and to call before any decision about DAPT withdrawal. The phone number was active from Monday to Friday from 9 am to 12 am. A study coordinator received the phone calls and, if deemed necessary, contacted one of the study group physicians to deal with patients’ requests;
6. two phone calls per month by the study coordinator to assess DAPT compliance and potential BARC 1 bleedings.

At one month, patients of both groups underwent the scheduled follow-up visit. Before the visit, one of the study coordinators collected the answers to the questionnaire regarding evidence of nuisance bleeding, their management, bleeding impact, information request (see S5 File) as well as EQ5D and EQ5D-VAS. Afterwards, patients in the interventional group received a 15 minutes visit (added to the usual one month follow-up visit) in which information regarding DAPT and nuisance bleeding were reinforced.

### Standard of care (control arm)

Patients randomized to the control arm (standard of care) received education from physicians regarding DAPT, as per routine discharge process In our practice, we use a discharge checklist for fellows or treating physicians (see S1 File for detailed description of the discharge process and S6 File for discharge checklist). The treating physician explained in detail to the patient and to his/her relatives the prescribed therapy (both antiplatelet and non-antiplatelet agents), emphasized the importance of compliance to the therapy, and underlined the main benefits and risks related to every prescribed drug. The duration of the talk was 10–15 minutes.

### Study endpoints

The primary endpoint is the one-month health-related quality of life assessed by the EuroQol-5 Dimension (EQ-5D) visual analog scale (VAS) score. Patients estimated their overall health status on a 20-cm VAS with the endpoints being “best imaginable health state” (score = 100) and “worst imaginable health state” (score = 0) [13]. The EQ-5D VAS is a quantitative measure of patients’ perceived health, a patient-centered health outcome. EQ-5D VAS was evaluated in all patients at baseline, after 1 and 6 months. The EQ-5D instrument at one and 6 months as well as the EQ-5D VAS at 6 months were used as secondary outcomes of interest. The EQ-5D instrument measures health status in 5 dimensions: mobility, self-care, usual activities, pain/discomfort, and anxiety/depression [13]. Each dimension is rated according to the following levels: i) no problems; ii) some problems; iii) extreme problems. It has been previously validated for patients with acute coronary syndrome [14]. Other secondary endpoints were ischemic (cardiovascular death, myocardial infarction, target lesion revascularization, re-PCI) and bleeding events (classified according to the BARC classification) and DAPT withdrawal. All “bleeding-related” events were evaluated as secondary endpoints (general practitioner visit, emergency room admission, hospitalization, red blood cell (RBC) transfusion). At the 6-month visit, patients were asked about their need of information regarding DAPT risks/benefits or nuisance bleedings (see S5 File for details). All clinical events were adjudicated by source documentation by physicians blinded to patients’ randomization (CT, MT).

### Follow-up

As per institutional practice, all patients underwent a follow-up visit at 1 and 6 months. During the visits EKG was recorded and laboratory tests were collected. If necessary, medical therapy was optimized and further tests were requested. Patient-reported bleeding and compliance to DAPT were assessed during follow-up visit at 1 and 6 months post-PCI by one of the study members (see S5 File for the detailed questionnaire administered to patients at 1 and 6 months). Patients’ adherence was monitored via self-reports. Bleeding events were classified according to BARC classification and were assessed at 1 and 6 months [15]. In particular, BARC 1 bleedings were defined as not actionable, and did not cause unscheduled visits or treatment. BARC 2 bleedings were defined as overt, actionable sign of hemorrhage that does not fit the criteria for type 3, 4, or 5 with at least one of the following criteria: requiring nonsurgical, medical intervention by a healthcare professional, leading to hospitalization or increased level of care, or prompting evaluation.

### Sample size

According to data from Amin et al. [3], we estimated a 1-month EQ-5D VAS mean value of 75±19. The study hypothesis was that counseling intervention could increase EQ-5D VAS by 8%. Consequently, according to two-sample t-test power calculation for continuous outcome superiority trial with Power 1-beta = 0.90, type I error rate alpha = 5% assuming 90% power and a 2-sided alpha of 0.05, the overall required sample size was a minimum of 422 patients (211 per group).The sample size was inflated to 448 patients (224 per group) to account for possible drops-out, losses to follow-up, and withdrawals of consent.

### Statistical analyses

Continuous data was tested for normal distribution with the Kolmogorov-Smirnov test. Normally distributed variables are presented as mean±standard deviation (SD) and were compared by t test. Otherwise, they are presented as median and interquartile range (IQR) and the Mann-Whitney U test was used. Categorical variables are summarized in terms of number and percentages and were compared by using Chi-square test. A 2-sided value of p<0.05 was considered significant. All analyses were performed with STATISTICA 8 (Statsoft Inc, Tulsa, Okla, USA).

## Results

The mean age of the study population was 69±12. More than 70% of patients were admitted for acute coronary syndrome (ACS) (Table A in S1 File). Most of the procedures (90%) were performed via radial access and half of the patients were discharged on DAPT with new P2Y12 inhibitors (Table A in S1 File). The two groups were comparable regarding all baseline and procedural characteristics (Table A in S1 File). There were no lost to follow-up, the follow-up duration was 6 months for all patients. All survived patients came to 1- and 6-months follow-up visits. Ischemic and bleeding events at 6 months did not differ between the two groups (Table 1). More than one third of the patients experienced a BARC 1 bleeding during the follow-up (Table 1). Of note, BARC 1 occurrence was significantly higher in patients treated with “new” P2Y12 inhibitors (ticagrelor or prasugrel) compared to those on clopidogrel (Table C in S1 File). EQ-5D VAS value at baseline was comparable between the two groups (64[51–74] in the control group vs. 63[50–76] in the interventional group, p = 0.9).

**Table 1:** Study endpoints.

	Control arm (n = 224)	Interventional arm (n = 224)	p
**Primary endpoint**			
EQ5D-VAS at 1 month, no.	73[64–80]	81[74–88]	p < 0.001
**Secondary endpoints at 6 months**			
EQ5D-VAS, no.	74 [65–81]	82[76–88]	p < 0.001
*Ischemic events*, *no*. *(%)*			
CV death	2 (1)	1 (0.4)	0.6
MI	4 (2)	4 (2)	1
TLR	7 (3)	6 (3)	0.8
Re-PCI	9 (4)	8 (4)	0.8
Definite/Probable ST,	2 (1)	1 (0.4)	0.6
*Bleeding events*, *no*. *(%)*			
BARC 1,	71 (32)	70 (31)	0.9
BARC 2,	14 (6)	11 (5)	0.5
BARC 3,	2 (1)	1 (0.4)	0.6
BARC 2–3,	15 (7)	12 (5)	0.6
BARC 5,	0 (0)	0 (0)	1
*Other secondary endpoints*, *no*. *(%)*			
DAPT withdrawal,	7 (3)	1 (0.4)	0.03
General practitioner visit,	12 (5)	10 (4)	0.7
Emergency room admission,	6 (3)	4 (2)	0.5
Hospitalization,	4 (2)	3 (1)	0.7
RBC transfusion,	2 (1)	1 (0.4)	0.6

EQ5D-VAS: EuroQol-5 Dimension visual analog scale. CV: CardioVascular. MI: myocardial infarction. TLR: target lesion revascularization. PCI: percutaneous coronary intervention. ST: stent thrombosis. BARC: Bleeding Academic Research Consortium. DAPT: dual antiplatelet therapy. RBC: red blood cells.

### Primary endpoint

**Fig 2:**
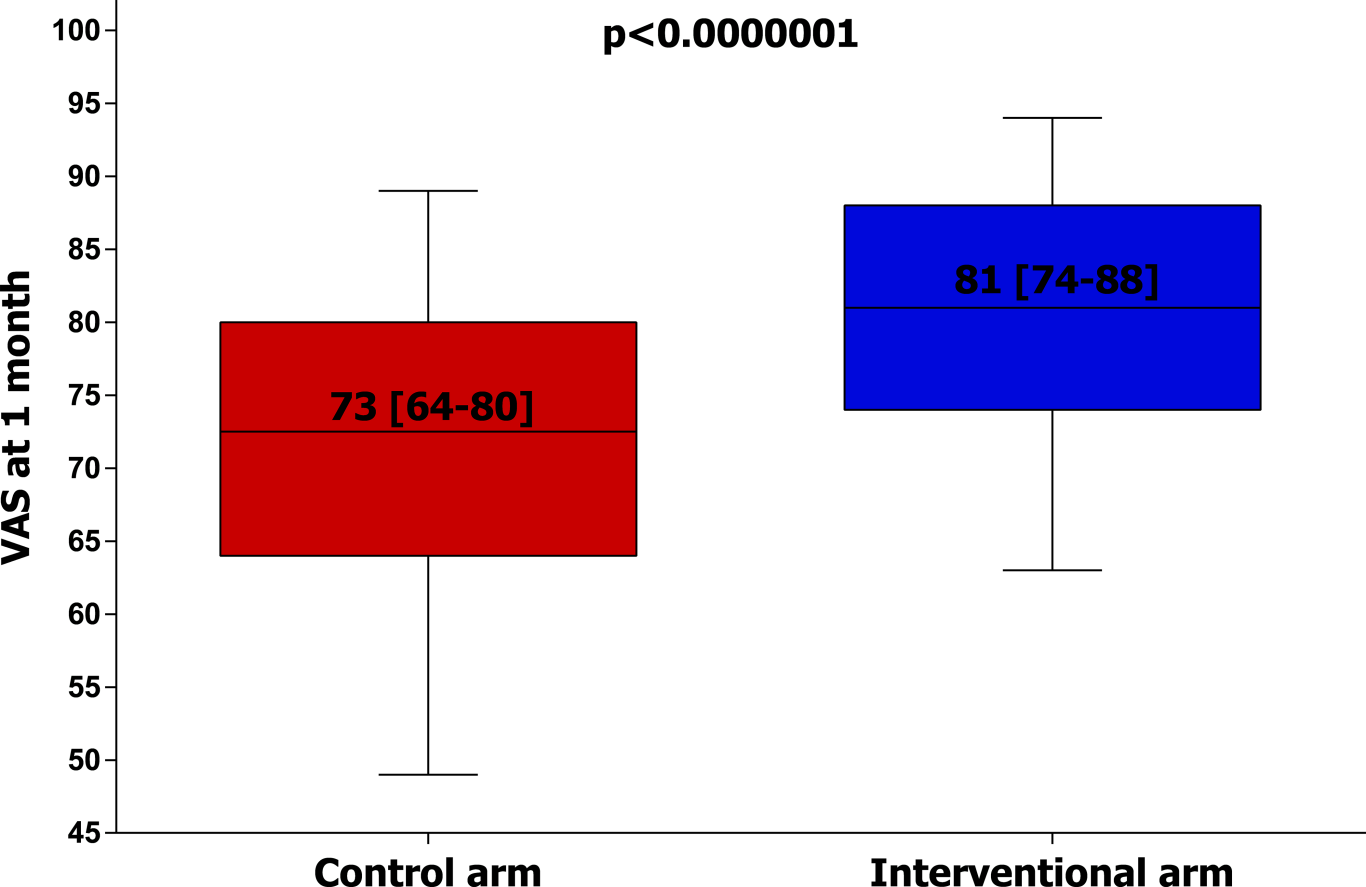
Primary endpoint: EQ5D-VAS at one-month. EQ5D-VAS values at one-month in patients of the two groups. The horizontal line shows the median value. The box showed the interquartile range. The vertical line shows the 10–90th percentile. EQ5D: EuroQol-5 Dimension. VAS: visual analog scale score.

The EQ5D-VAS value at 1 month was significantly higher in the interventional arm compared to control arm (81[74–88] vs. 73[64–80], p < 0.001, Table 1 and Fig 2).

### Quality of life at 1 and 6 months

At 6 months, patients in the interventional arm maintained a significantly higher EQ5D-VAS value compared to patients of the control arm (82[76–88] vs. 74[65–81], p < 0.001, Table 1). Patients in the interventional arm had also significantly lower pain/discomfort and anxiety/depression at the EQ-5D both at 1 and 6 months (p<0.01 and p = 0.01 respectively, Table 2). On the contrary, there were no differences regarding mobility, self-care and usual activities between the two groups at either 1 or 6 months (Table 2 and Figure A in S1 File).

**Table 2:** EQ-5D results at one and six months.

EQ-5D	1 month			6 months		
	Control arm (n = 224)	Interventional arm (n = 224)	p	Control arm (n = 222)	Interventional arm (n = 223)	p
*Pain/discomfort*, *no*. *(%)*			0.02			<0.01
I have no pain/discomfort	168 (75)	206 (92)		150 (68)	203 (91)	
I have moderate pain/discomfort	51 (23)	16 (7)		63 (28)	18 (8)	
I am in extreme pain/discomfort	5 (2)	2 (1)		9 (4)	2 (1)	
*Anxiety/depression*, *no*. *(%)*			0.01			0.01
I am not anxious or depressed	172 (77)	211 (94)		168 (76)	207 (93)	
I am moderately anxious or depressed	46 (21)	12 (5)		48 (22)	14 (6)	
I am extremely anxious or depressed	6 (2)	1 (1)		6 (2)	2 (1)	
*Mobility*, *no*. *(%)*			1			1
I have no problems in walking about	191 (85)	193 (86)		185 (83)	186 (83)	
I have some problems in walking about	33 (15)	31 (14)		39 (17)	38 (17)	
I am confined to bed	0 (0)	0 (0)		0 (0)	0 (0)	
*Self-Care*, *no*. *(%)*			1			0.9
I have no problems with self-care	217 (97)	218 (97)		213 (96)	216 (97)	
I have some problems washing or dressing myself	7 (3)	6 (3)		8 (4)	7 (3)	
I am unable to wash or dress myself	0 (0)	0 (0)		1 (0)	0 (0)	
*Usual Activities (e*.*g*. *work*, *study*, *housework*, *family or leisure activities)*, *no*. *(%)*			0.3			0.9
I have no problems with performing my usual activities	199 (89)	201 (90)		199 (90)	202 (91)	
I have some problems with performing my usual activities	23 (10)	22 (10)		22 (10)	21 (9)	
I am unable to perform my usual activities	2 (1)	1 (0)		1 (0)	0 (0)	

EQ5D: EuroQol-5 Dimension.

### Impact of “New” P2Y12 inhibitors on nuisance bleeding and quality of life

Patients treated with newer P2Y12 inhibitors (either ticagrelor or prasugrel) experienced significantly more nuisance bleedings if compared to those on clopidogrel (25 vs. 38%, p = 0.003, see Figure A in S1 File). The difference in EQ-5D VAS value at 1- and 6-months between the two groups was consistent irrespectively from the P2Y12 inhibitor utilized (Figure A in S1 File).

### DAPT withdrawal and bleeding-related events

Patients in the control arm withdrew DAPT significantly more compared to those in the interventional arm (7 (3%) vs. 1 (0.4%), p = 0.03). The reasons for DAPT withdrawal were: occurrence of BARC 3 bleeding (1 patient per group (0.4%)); occurrence of BARC 2 bleeding (1 patient in the control group (0.4%)); no other compelling reason beside nuisance bleeding occurrence (5 patients in the control group). There were no significant differences between the control and interventional arm regarding rate of general practitioner visit, emergency room access and hospitalization (12 (5%) vs. 10 (4%), p = 0.7, 6 (3%) vs. 4 (2%), p = 0.5, 4 (2%) vs. 3 (1%), p = 0.7, respectively, Table 1). Equally, we found no differences in the number of red blood cells transfusions between the two groups (2 (1%) vs. 1 (0.4%), p = 0.6, Table 1).

### Information request

Throughout the 6 months of treatment, patients in the control arm searched for information on DAPT risks and benefits and/or nuisance bleeding more frequently than patients in the interventional arm (178 (79%) vs. 19 (8%), p < 0.001, Table C in S1 File). Sixty-seven percent of patients in the control arm requested information on nuisance bleedings and 46% of them asked to the general practitioner information on DAPT risks and benefits (both p < 0.001 vs. study group, Table C in S1 File). Of note, 61% of the patients in the control arm asked information to friends or relatives (p < 0.001 vs. interventional group, Table C in S1 File).

## Discussion

The BATMAN study is the first randomized trial addressing nuisance bleedings in patients on DAPT. Our findings are straight-forward and clinically relevant: i) incidence of nuisance bleeding in a contemporary population of patients on DAPT is above 30%; ii) impact on quality of life of nuisance bleeding is relevant and can be reduced through a counseling program directed to patients and their relatives; iii) DAPT withdrawal was significantly higher in the control group compared to the interventional group.

In our study, incidence of nuisance (BARC 1) bleeding was above 30% during a 6 months follow-up (Table 1). Interestingly, BARC 1 occurred significantly more in patients receiving “new” P2Y12 inhibitors (ticagrelor or prasugrel) if compared to those receiving clopidogrel (see Table B in S1 File). Our results confirm that the incidence of nuisance bleeding increases proportionally with intensity and duration of DAPT. Beyond being frequent, nuisance bleeding significantly impacts on patient’s quality of life [3,10]. Previous studies by Amin et al [3,10] pointed out the critical need to further investigate the role of patient’s education on nuisance bleedings to implement quality of life and DAPT adherence. Our study embraced and confirmed their hypothesis showing that the systematic use of a counseling strategy on DAPT and nuisance bleedings was able to improve quality of life assessed by EQ-5D VAS at 1 and 6 months (Table 1 and Fig 2) and to reduce the rate of pain/discomfort and anxiety/depression at the EQ-5D at 1 and 6 months (Table 2). Our study shows no “late catch-up” phenomenon, as the magnitude of the quality of life improvement was maintained at 6 months. The “wide-spectrum” approach directed to patient, relatives, and general practitioner as well as the reiteration of the message are probably the key aspects that allowed us to maintain the benefit over time. The benefit was consistent with all antiplatelet therapeutic regimens (Figure A in S1 File). At the same time, the improvement in the EQ-5D VAS was numerically greater in patients treated with “new” P2Y12 inhibitors, probably because of the higher incidence of nuisance bleedings consequent to stronger platelet inhibition (Table B in S1 File). In a previous study, 75% of DAPT withdrawals were due to nuisance bleeding [2], while in another one nuisance bleeding was associated with clopidogrel cessation in 11% of patients [1]. On the other hand, patients receiving specific education regarding the need of antiplatelet therapy adherence are less likely to withdraw DAPT [16,17]. The dramatic consequences of DAPT withdrawal are well-known and widely demonstrated [18]. We found a significant reduction of DAPT withdrawal in patients receiving the counseling program compared to those in the control arm (Table 1). Of note, in 5 out of 7 patients who withdrew DAPT in the control group we found no compelling clinical reason for drug discontinuation except for nuisance bleeding occurrence. Moreover, we found no difference between the two groups regarding general practitioner visit or emergency room access, suggesting that patients not receiving adequate information tend to suspend DAPT autonomously, without consulting any physician. It is important to point out that the present study was not primarily designed to detect a difference for DAPT withdrawal and not powered for clinical endpoints. Thus, our results on DAPT withdrawal and the possible clinical implication of the counseling program are “hypothesis-generating” and should be confirmed in adequately powered trials.

Patients’ need to be informed on nuisance bleeding is demonstrated by the fact that almost 80% of patients in the control arm looked for it with a highly significant difference compared to the interventional arm (Table C in S1 File). Furthermore, reaching this information did not translate in a quality of life improvement or in a reduction of DAPT withdrawal in the control arm. We can infer that the “quality” of information received by patients is pivotal in determining quality of life and adherence to therapy. Information given by relatives/friends may not be enough to reassure patients regarding DAPT and nuisance bleedings. Moreover, the increasing utilization of internet as a source of medical information may generate worries in the patient, especially related to the overrepresentation of minor side effects (as nuisance bleedings are) and may eventually lead to “self”-drug discontinuation.

The essential message of our study is that it is crucial to inform patients regarding the high likelihood of nuisance bleeding occurrence and to reassure them about the need to continue DAPT independently from nuisance bleeding occurrence, rather than the adoption “tout-court” of our counselling program. This could relevant not only for the Cardiologist who discharges the patient, but also for the General Practitioner as well as for Outpatient Cardiologist who may deal with patients on DAPT. Our program highlights the importance of the reiteration of counseling as well as the active involvement of relatives in order to make them a further vehicle of information and an “ally” in the avoidance of drug discontinuation. Our results are novel and potentially impactful on everyday clinical practice, but the present study represents a first preliminary evidence of a possible benefit related to a counseling program that should be confirmed in a larger study with clinical endpoints. Moreover, the difference in quality of life given by the counseling program is huge and again it should be confirmed in broader populations. The major limitations of our study are represented by the single-center enrollment with an open design and by the lack of power for hard clinical endpoints. It would have been probably more appropriate to choose the primary endpoint at 6 months. However, we based our choice on available data [3] and the difference between the two groups was confirmed at 6 months. The external validity of our findings should be confirmed in broader contexts and in different healthcare systems. Further studies are on demand to confirm our results with longer follow-up in patients on long-term DAPT [7, 9]. Moreover, we excluded patients with bleeding diathesis history or with indication to anticoagulant therapy to obtain a homogeneous population. However, these patients are those with the theoretical major benefit from a counseling program on nuisance bleedings. Finally, our program has a non-negligible burden in terms of extra-time needed per patient and might not be cost-effective in some health systems. The economic impact of this extra-time is highly variable on the basis of the institutional organization. It is interesting to note that also new technologies in interventional cardiology focused great part of their benefices on quality of life, such as coronary sinus reducer (Reducer, Neovasc) [19] and Bioresorbable Vascular Scaffold (NCT02173379).

## Conclusions

The systematic utilization of a counseling program increased quality of life and reduced DAPT withdrawal rate. Our study addressed one of the current unmet clinical needs regarding DAPT and the proposed program could become standard of care in patients with indication to DAPT after stent implantation. Adequately powered randomized trials are on demand to investigate the impact of our counseling program on hard endpoints.

## Supporting information

S1 File Extensive methods; supplementary tables and figure. (DOCX)

S2 File Pre-discharge powerpoint presentation: DAPT & bleedings. (PDF)

S3 File Antiplatelet drugs and minor bleedings: Patient’s guide. (PDF)

S4 File Antiplatelet drugs and minor bleedings: General practitioner’s disclosure. (PDF)

S5 File Patient’s questionnaire on impact of nuisance bleeding on quality of life. (PDF)

S6 File Predischarge checklist. (PDF)

S7 File Protocol (English version). (DOCX)

S8 File Protocol (Italian version). (DOCX)

S9 File CONSORT checklist. (PDF)

S10 File Portion of study database. (XLSX)

## Abbreviations

DAPT: dual antiplatelet therapy
PCI: percutaneous coronary intervention
BARC: Bleeding Academic Research Consortium
DES: drug eluting stent
EQ-5D VAS: EuroQol-5 Dimension visual analog scale
RBC: red blood cell
SD: standard deviation
IQR: interquartile range
CI: confidence interval
ACS: acute coronary syndrome
