# Supplementary material for: A counseling program on nuisance bleeding improves quality of life in patients on dual antiplatelet therapy: A randomized controlled trial
Source: PLoS One. 2017 Aug 23;12(8):e0182124. doi: 10.1371/journal.pone.0182124 (PMC5568410; doi:10.1371/journal.pone.0182124)
Supplement: S1 File — (DOCX) [file pone.0182124.s001.docx]

**SUPPLEMENTARY DATA.**

Page 2: Extensive Methods.

Page 6: Table A. Baseline and procedural characteristics of the study population.

Page 7: Table B. BARC bleedings stratified according P2Y12 inhibitor.

Page 8. Table C. Patients requiring further information regarding dual antiplatelet therapy risks and benefits.

Page 9. Figure A EQ-5D VAS values over time stratified according P2Y12 inhibitor.

**Extensive methods**

**Routine discharge process**

Patients randomized to the control arm (standard of care) received education from physicians regarding dual antiplatelet therapy (DAPT), as per routine discharge process. In our practice, we use a discharge checklist for fellows or treating physicians (see Online Data Supplement 6). The checklist includes: predischarge EKG acquisition, DAPT prescription, explanation of DAPT risks and benefits, follow-up visit scheduling, explanation of heart failure management (with scheduled follow-up visit to the Heart Failure center if indicated), diabetes management (with scheduled follow-up visit to the Diabetes center if indicated), lifestyle adjustments . The discharging physician had to check each box labeled and to give the filled form to the study coordinator before patients’ discharge. In addition, copy of discharge letter was electronically sent to patient’s general practitioner.

**Study intervention**

Patients randomized to the interventional arm received:

1. a 15-minutes meeting with a member of the research team 24 hours prior discharge. During this visit, a core set of DAPT risks were addressed. DAPT advantages and side effects were described. The importance of compliance and the correct management of side effects (especially BARC 1 bleedings) were discussed;
2. a 15-minutes meeting with a member of the research team with a family member living near/with the patient addressing the same issues: DAPT advantages and side effects, importance of compliance, side effect management;
3. a brochure describing DAPT advantages, side effects, and management;
4. a brochure for the patient’s general practitioner aimed at presenting DAPT rationale and management. Contemporaneously, study investigators directly contacted by phone and/or by email the patient’s general practitioner to describe and explain the same topics and the clinical problem of his/her patient;
5. a phone number to further discuss potential side effects of DAPT and to contact before any decision about DAPT withdrawal. The phone number was active from Monday to Friday from 9 am to 12 am. A study coordinator received the phone calls and, if deemed necessary, contacted one of the study group physicians to deal with patients’ requests;
6. two phone calls per month by the study coordinator to assess DAPT compliance and potential BARC 1 bleedings.

At one month, patients of both groups underwent the scheduled follow-up visit. Before the visit, one of the study coordinators collected the answers to the questionnaire regarding evidence of nuisance bleedings, their management, bleeding impact and information request as well as EQ5D and EQ5D-VAS. Afterwards, patients in the interventional group received a 15 minutes visit (added to the usual one month follow-up visit) in which information regarding DAPT and nuisance bleeding were reinforced.

Notably, the discharging physician was trained to use very simple concepts and words, avoiding technical language (i.e.: heart attack instead of myocardial infarction, blood vomiting instead of hematemesis). Moreover, the member of the study team used a PowerPoint presentation with information regarding DAPT, usual activities and management of nuisance and alarming bleedings along with iconographic images in order to help patients to discriminate between the two types of bleeding (see Online Data Supplement 2).

During both visits, a core set of DAPT advantages and risks were addressed. Below we detail the main topics covered (see Online Data Supplement 2 and 3 for detailed description).

**Visits topics**

1. **DAPT advantages**

The physician explained to the patient that DAPT is able to dramatically reduce his/her risk of death and future cardiac events, namely re-infarction and re-percutaneous coronary interventions (PCI). In particular, we described to the patient the dramatic effect of a re-infarction in the same area already treated with a stent (stent thrombosis) and told him/her that the first cause of stent thrombosis is DAPT withdrawal (see Online Data Supplement 2 and 3 for detailed description).

1. **DAPT side effects**

The patient was informed that, because of its intrinsic mechanism of action, the main DAPT side effect is bleeding. Moreover, the physician explained to the patient that there are two different type of bleeding DAPT-related. The first is a nuisance bleeding: epistaxis, gum bleeding, small superficial bleeding, bruises. This kind of bleeding is not alarming; in fact it is an indirect sign of DAPT efficacy. The second type of bleeding is any conspicuous bleeding, as vomiting of blood or blood in stools. The management of each of these bleedings has been illustrated in detail (see Online Data Supplement 2 and 3 for detailed description).

1. **Importance of compliance**

The physician clearly stated that the first cause of stent thrombosis (and then of a further myocardial infarction) is the withdrawal of one or both antiplatelet agents. The patient was informed that the prognosis after a stent thrombosis is very poor even reopening of the vessel with PCI. Thus, the patient has not to withdraw any antiplatelet treatment on his/her own and without the consent of a cardiologist (see Online Data Supplement 2 and 3 for detailed description).

1. **Management of side effects (especially BARC 1 bleedings)**

The physician told the patient that he/she could restart his/her usual activities (i.e.: gardening, sport activities, shaving, cooking…). However, he/she has to know that small bleedings and/or bruising require longer time to stop because of DAPT. At the same time, patients were reassured that this is normal and that they have not to be worried. The physician described the management of every type of bleeding. For nuisance bleedings (bruising, nose bleed, gum bleed, petechiae, subconjunctival bleeding) the patient has nothing to do and should not worry. For bleedings such as melena or blood traces in spit, he/she should call his/her general practitioner. If the patient has hematemesis, he/she has to immediately call the emergency number (see Online Data Supplement 2 and 3 for detailed description).

**Patients’ brochure**

All patients in the study groups received a brochure with the same content covered during the visits. The aim of the brochure was both to reinforce the message and to further elucidate DAPT benefits and risk (see Online Data Supplement 3).

**General practitioner’s brochure**

All patients in the study group received a brochure for their general practitioner (see Online Data Supplement 4). The aim of this brochure was mainly to help general practitioner in the management of DAPT side effects. Moreover, other topics such as DAPT duration according stent and clinical presentation, thrombotic and bleeding risk assessment and triple therapy were covered.

**TABLES.**

**Table A. Baseline and procedural characteristics of the study population.**

|  | **Control arm**  **(n=224)** | **Interventional arm**  **(n=224)** | **p** |
| --- | --- | --- | --- |
|  |  |  |  |
| Age, yrs | 70±12 | 68±12 | 0.2 |
| Men, no. (%) | 179 (79) | 185 (83) | 0.5 |
|  |  |  |  |
| **Cardiovascular risk factors, no. (%)** |  |  |  |
| Hypertension, | 134 (60) | 131 (58) | 0.8 |
| Dyslipidemia, | 88 (39) | 76 (34) | 0.2 |
| Diabetes mellitus, | 53 (24) | 47 (21) | 0.5 |
| Current smoker, | 45 (20) | 45 (20) | 1 |
| Family history of CAD, | 67 (30) | 65 (29) | 0.9 |
| BMI (kg/m2) | 27±5 | 27±4 | 0.9 |
|  |  |  |  |
| **Medical history, no. (%)** |  |  |  |
| Myocardial infarction, | 43 (19) | 41 (18) | 0.8 |
| PCI, | 38 (17) | 34 (15) | 0.6 |
| CABG, | 19 (8) | 14 (6) | 0.4 |
|  |  |  |  |
| **Clinical presentation, no. (%)** |  |  | 1 |
| STEMI, | 67 (30) | 68 (30) |  |
| NSTEMI, | 62 (28) | 63 (28) |  |
| UA, | 35 (15) | 33 (15) |  |
| SCAD, | 60 (27) | 60 (27) |  |
|  |  |  |  |
| **Clinical data** |  |  |  |
| LVEF, %, | 50±11 | 49±11 | 0.8 |
| Severe CKD, no. (%), | 10 (4) | 8 (4) | 0.6 |
| COPD, no. (%), | 6 (3) | 6 (3) | 1 |
|  |  |  |  |
| **P2Y12 inhibitor at discharge, no. (%)** |  |  | 0.8 |
| Clopidogrel, | 110 (49) | 115 (51) |  |
| Prasugrel, | 6 (3) | 8 (4) |  |
| Ticagrelor | 108 (48) | 101 (45) |  |
|  |  |  |  |
| **Procedural characteristics** |  |  |  |
| *Number of treated vessels, no. (%)* |  |  | *0.9* |
| 1, | 160 (71) | 162 (72) |  |
| 2, | 52 (23) | 48 (21) |  |
| 3, | 12 (5) | 14 (7) |  |
| Radial access, no. (%), | 197 (88) | 207 (92) | 0.1 |
|  |  |  |  |

CAD: coronary artery disease. BMI: body mass index. PCI: percutaneous coronary intervention. CABG: coronary artery bypass graft. STEMI: ST-elevated myocardial infarction. NSTEMI: no ST-elevated myocardial infarction. UA: unstable angina. SCAD: stable coronary artery disease. LVEF: left ventricular ejection fraction. CKD: chronic kidney disease. COPD: chronic obstructive pulmonary disease.

**Table B. BARC bleedings stratified according P2Y12 inhibitor.**

|  | **Clopidogrel**  **(n=225)** | **Ticagrelor or Prasugrel**  **(n=223)** | **p** |
| --- | --- | --- | --- |
|  |  |  |  |
| BARC 1, no. (%) | 56 (25) | 85 (38) | 0.003 |
| BARC 2, no. (%) | 12 (5) | 13 (6) | 0.8 |
| BARC 3, no. (%) | 1 (0.4) | 2 (1) | 0.6 |
|  |  |  |  |

BARC: Bleeding Academic Research Consortium.

**Table C. Patients requiring further information regarding dual antiplatelet therapy risks and benefits.**

|  | **Control arm**  **(n=224)** | **Interventional arm**  **(n=224)** | **p** |
| --- | --- | --- | --- |
|  |  |  |  |
| *Patients requiring further information, no. (%)* | *178 (79)* | *19 (8)* | *<0.00001* |
|  |  |  |  |
| Info regarding nuisance bleedings, no. (%) | 149 (67) | 8 (4) | <0.00001 |
| General Practitioner, no. (%) | 103 (46) | 2 (1) | <0.00001 |
| Friends/Relatives, no. (%) | 137 (61) | 3 (1) | <0.00001 |
| Private Cardiologist, no. (%) | 36 (16) | 4 (2) | <0.00001 |
| Internet, no. (%) | 42 (19) | 16 (7) | 0.0003 |
|  |  |  |  |

**Figure A. EQ-5D VAS values over time stratified according P2Y12 inhibitor.**

A: EQ-5D VAS values in the whole population. B: EQ-5D VAS values in patients on clopidogrel. C: EQ-5D VAS values in patients on ticagrelor or prasugrel.


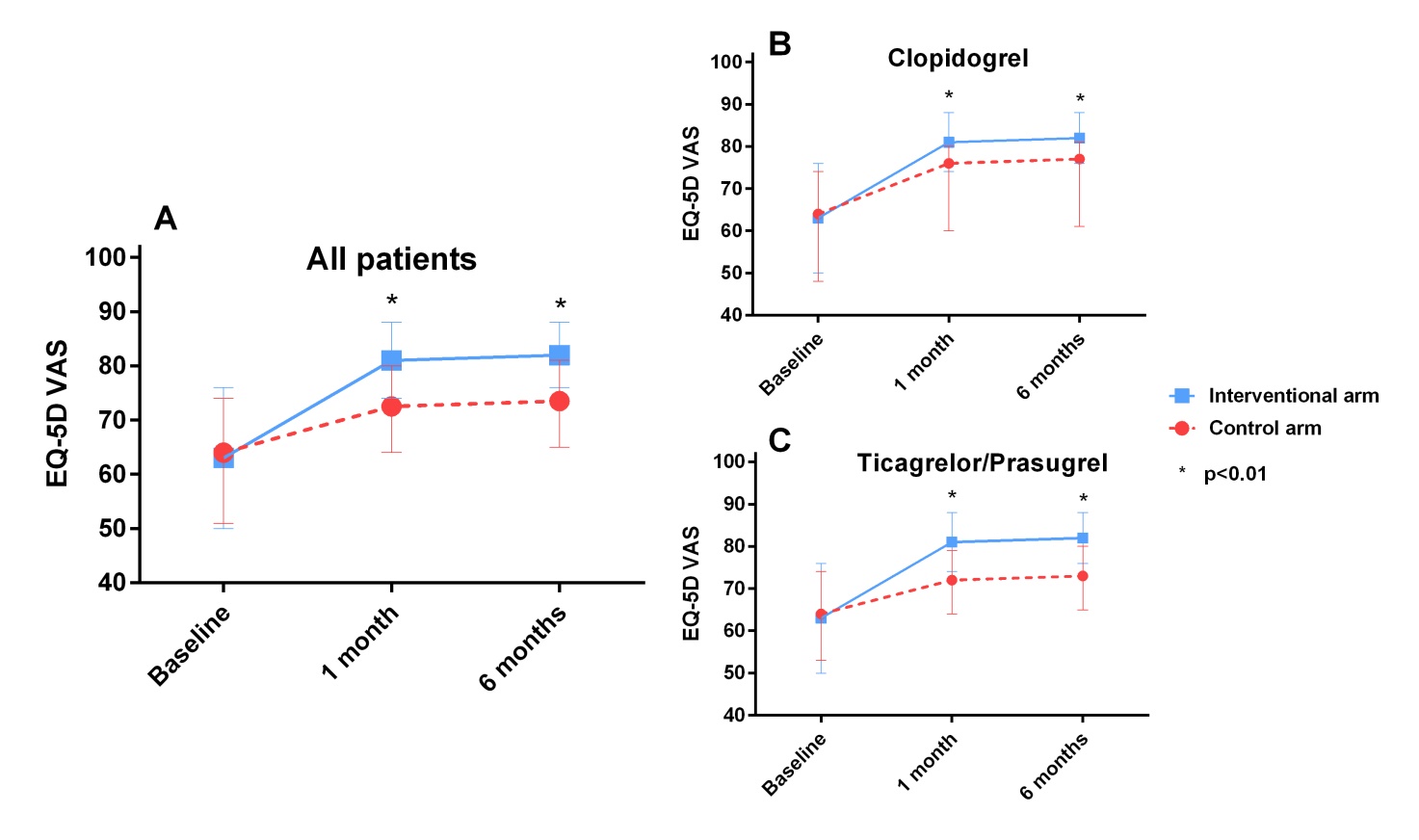


EQ5D: EuroQol-5 Dimension. VAS: visual analog scale score.
