# Supplementary material for: A counseling program on nuisance bleeding improves quality of life in patients on dual antiplatelet therapy: A randomized controlled trial
Source: PLoS One. 2017 Aug 23;12(8):e0182124. doi: 10.1371/journal.pone.0182124 (PMC5568410; doi:10.1371/journal.pone.0182124)
Supplement: S2 File — (PDF) [file pone.0182124.s002.pdf]

Pre-discharge presentation: DAPT & bleedings

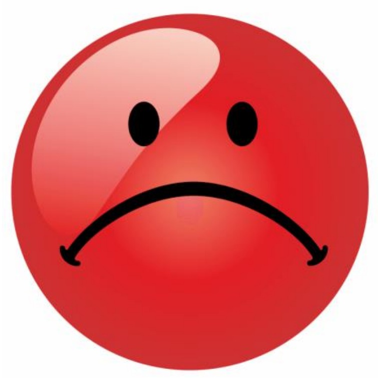

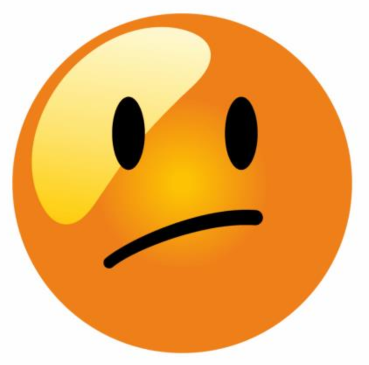

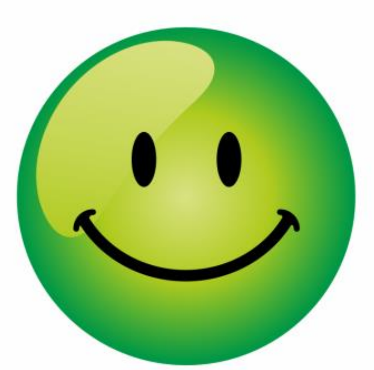

BATMAN trial

NCT02554006

UNIVERSITÀ DEGLI STUDI DI FERRARA
- EX LABORE FRUCTUS -

Why do I have to take aspirin + clopidogrel/prasugrel/ticagrelor?

↓ Mortality ↓ Infarction ↓ Stent thrombosis

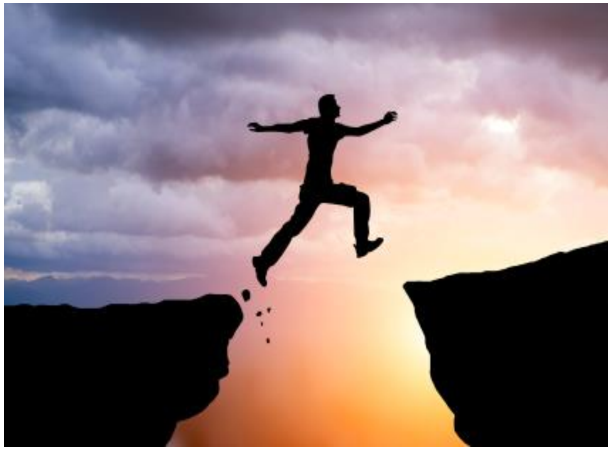

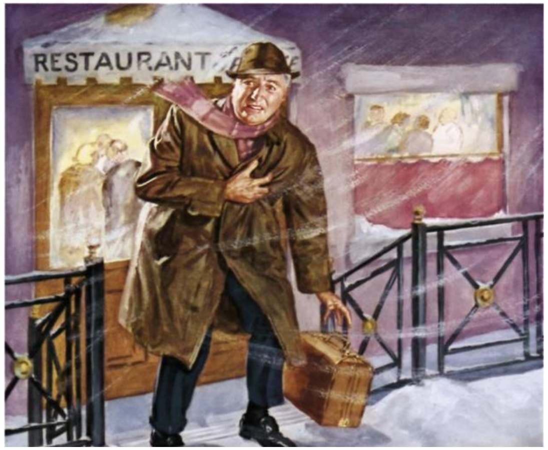

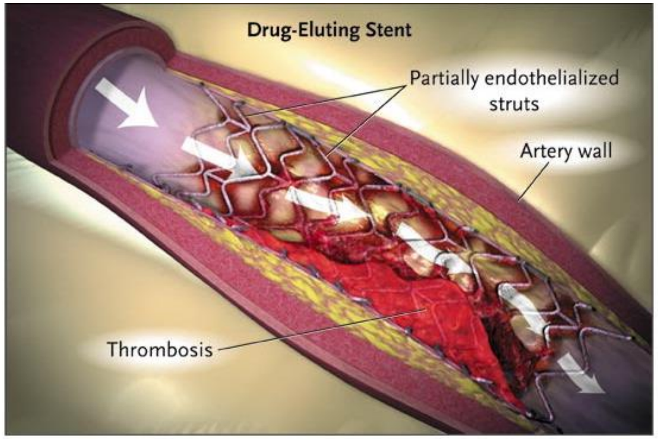

Can I restart my usual activities?

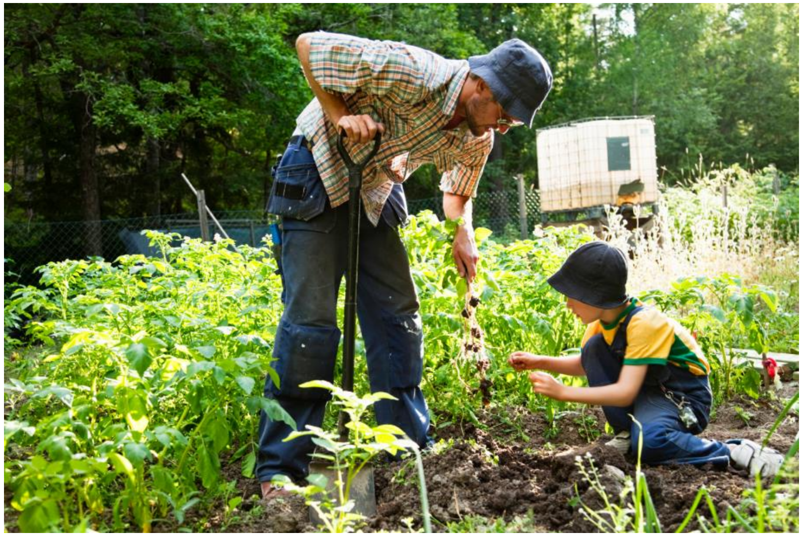

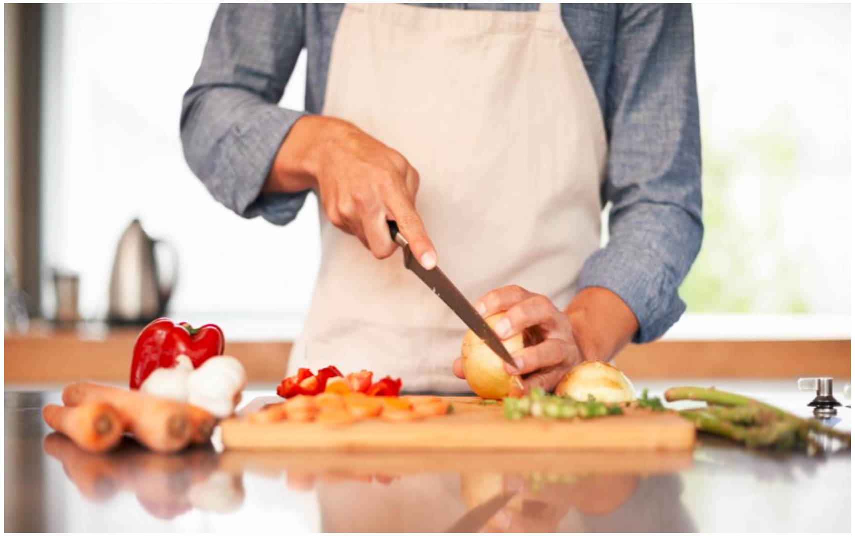

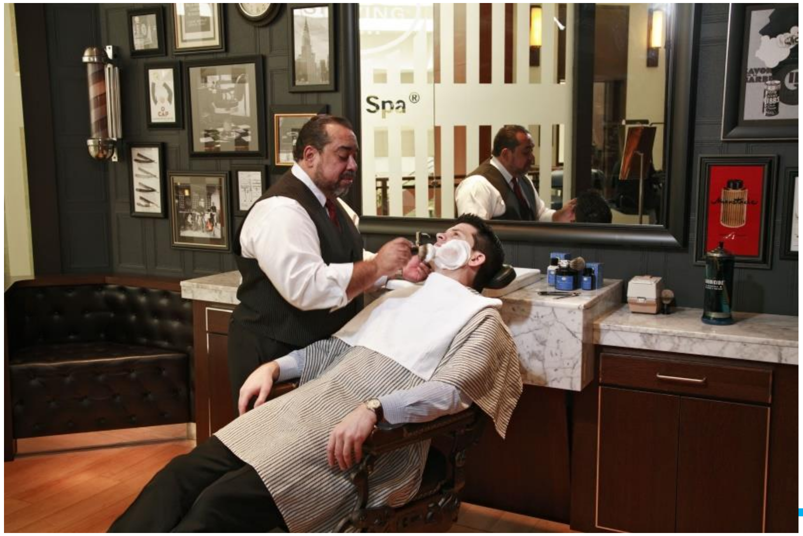

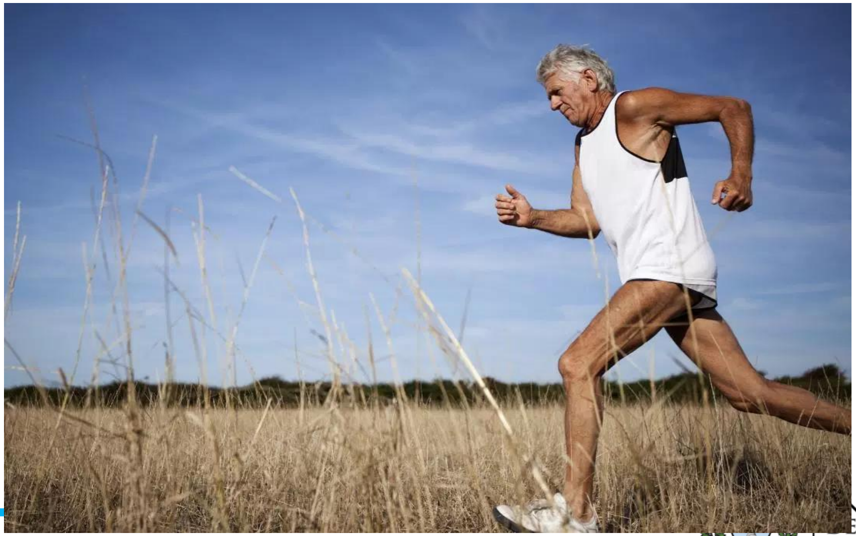

Can I restart my usual activities?

Yes, but remember that because of them:

- You can experience small bleeding or bruising
- Bleedings require longer time to stop: **it is normal, do not worry!!!!**

Bruising

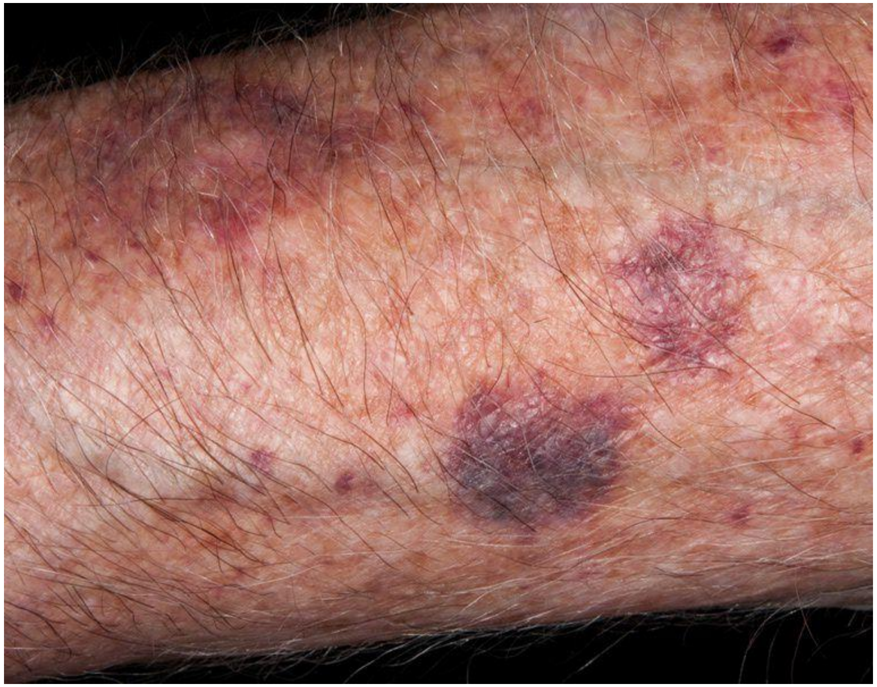

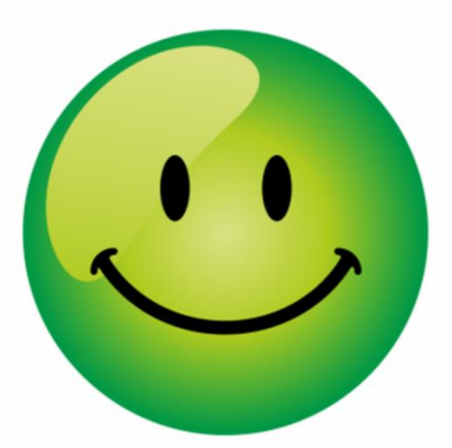

WHAT TO DO?

NOTHING

Nose bleed

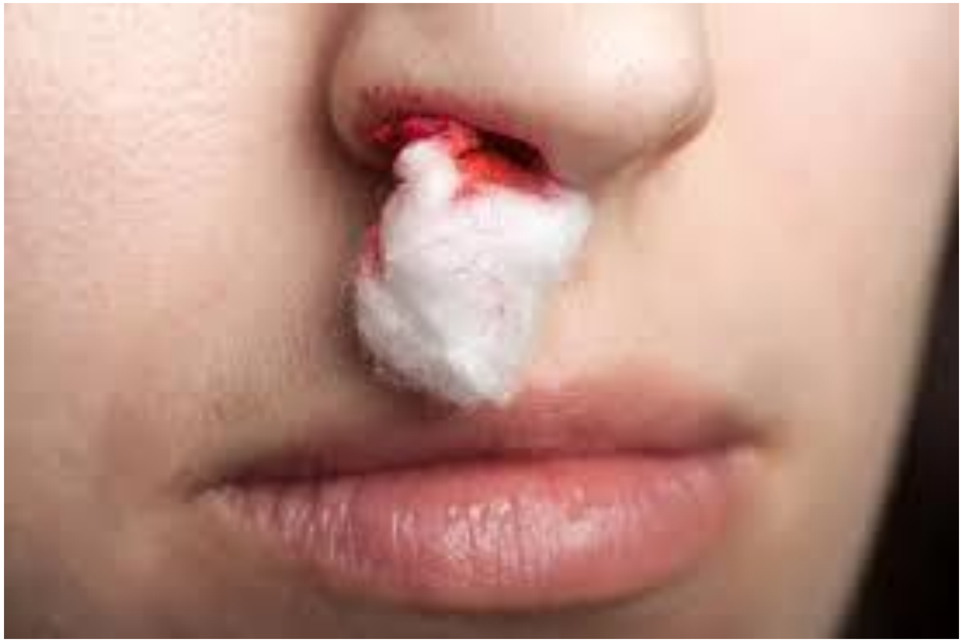

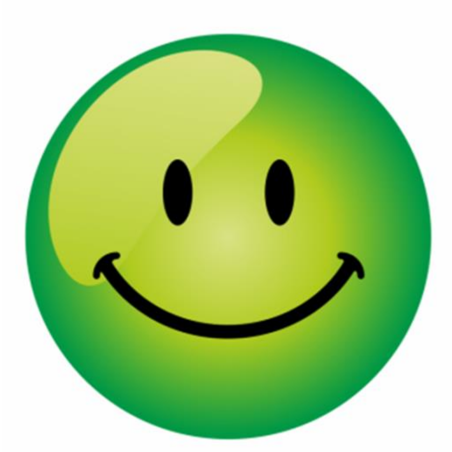

WHAT TO DO?

NOTHING

Gum bleed

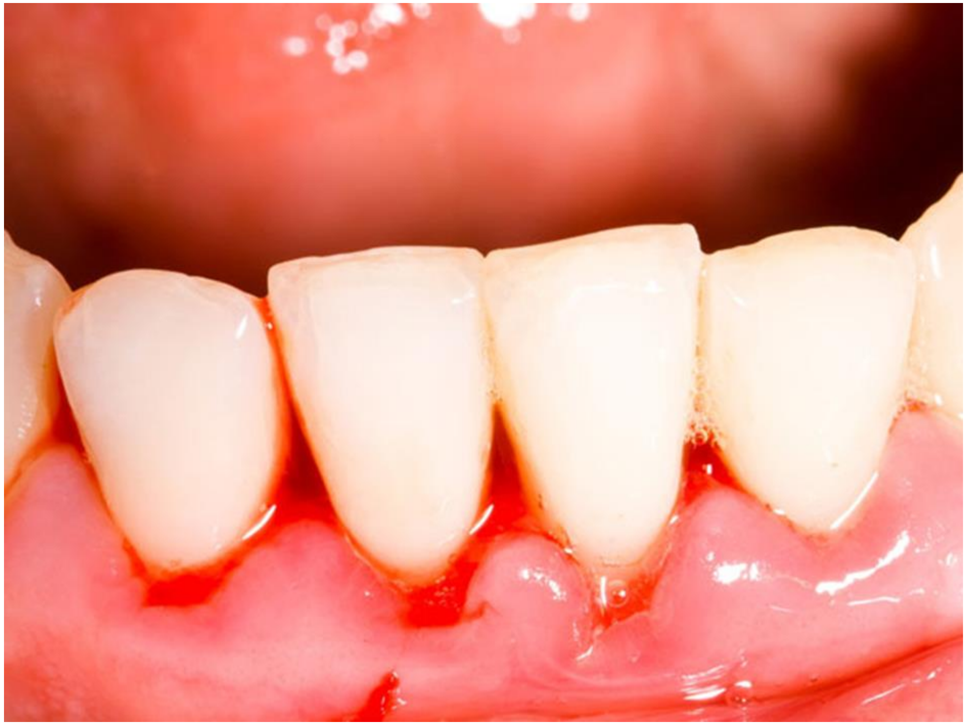

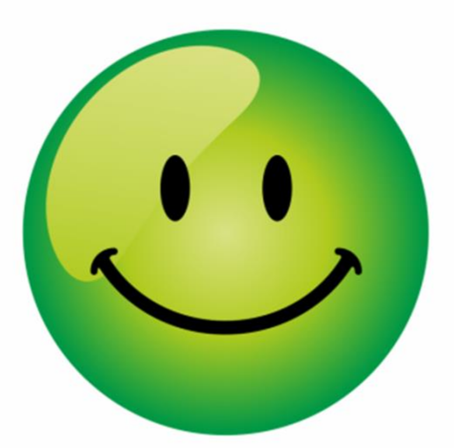

WHAT TO DO?

NOTHING

Small red spots

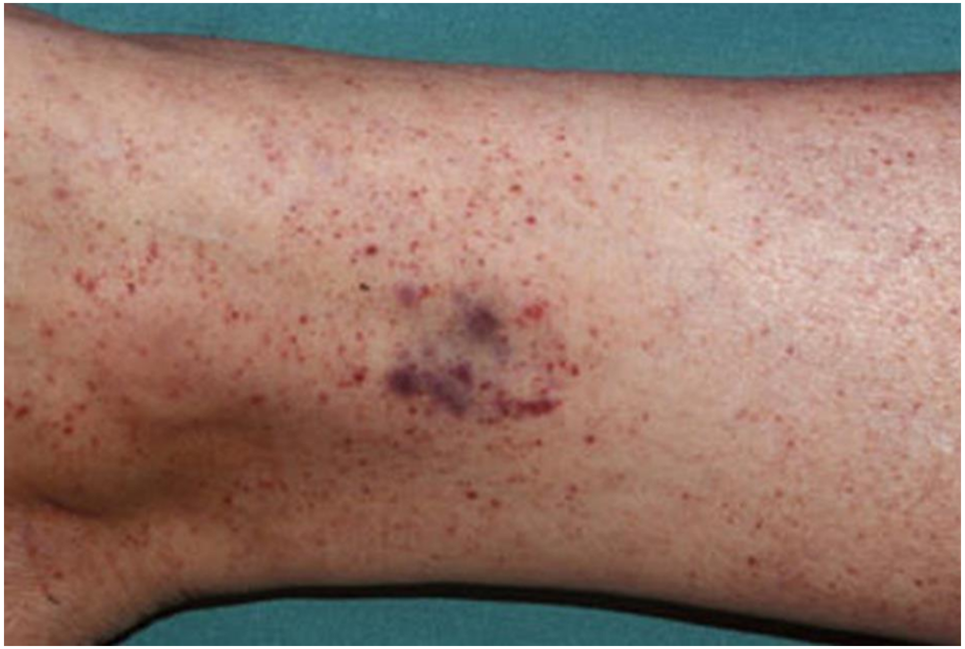

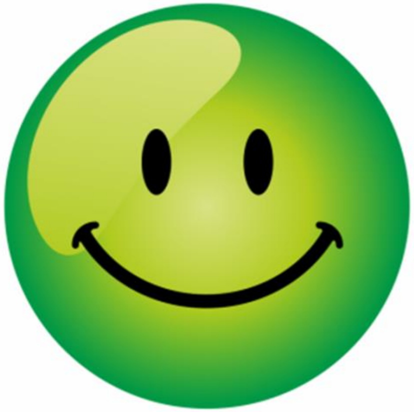

WHAT TO DO?

NOTHING

Subconjunctival bleed

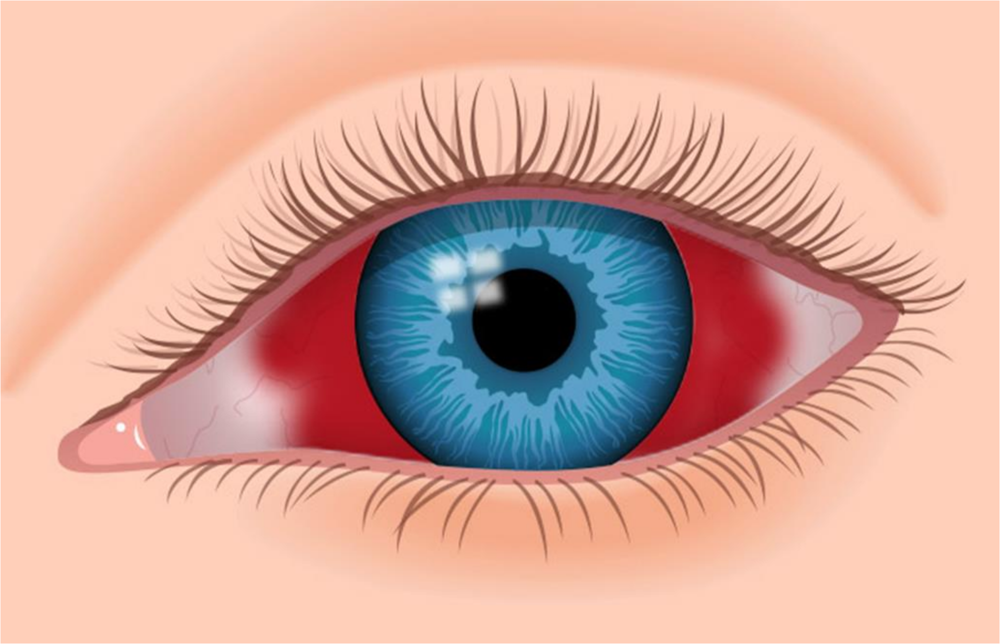

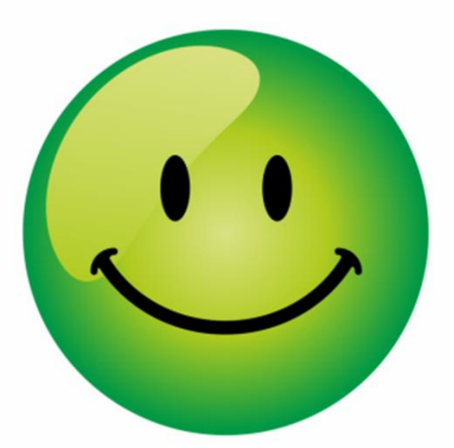

WHAT TO DO?

NOTHING

Black feces

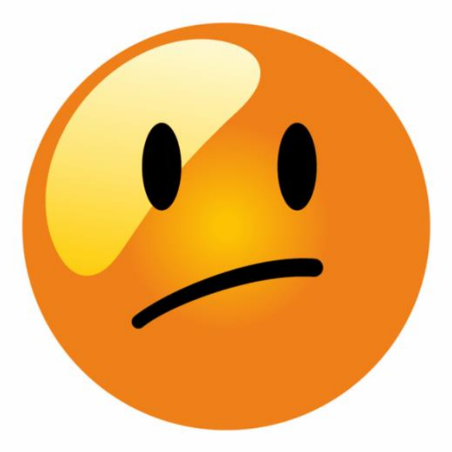

WHAT TO DO?

CALL YOUR DOCTOR

Small traces of blood in stools

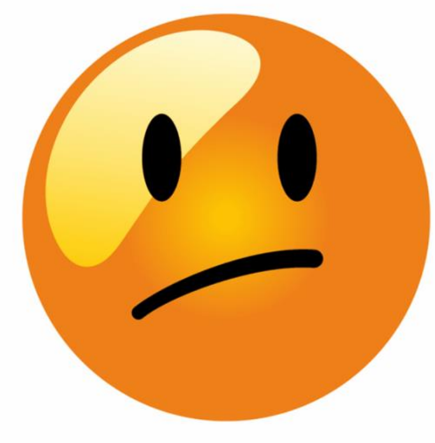

WHAT TO DO?

CALL YOUR DOCTOR

Red urine

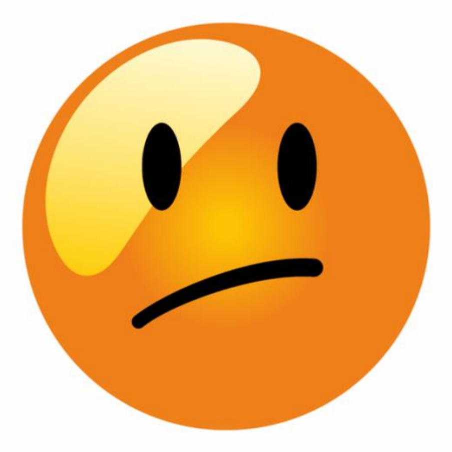

WHAT TO DO?

CALL YOUR DOCTOR

Blood traces in sputum

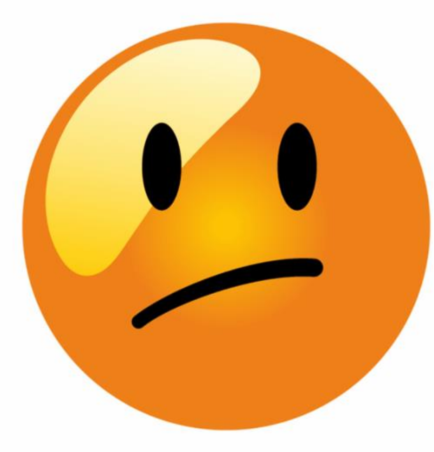

WHAT TO DO?

CALL YOUR DOCTOR

UNIVERSITÀ DEGLI STUDI DI FERRARA
- EX LABORE FRUCTUS -

Blood vomiting

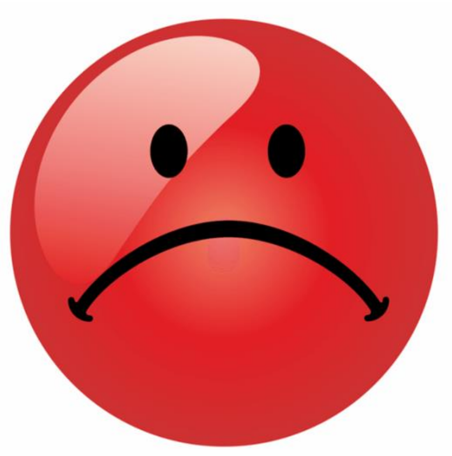

WHAT TO DO?

CALL EMERGENCY NUMBER

REMEMBER:

NEVER EVER WITHDRAW

1. ASPIRIN
2. CLOPIDOGREL (*PLAVIX*)
3. TICAGRELOR (*BRILIQUE*)
4. PRASUGREL (*EFIENT*)

WITHOUT CALLING US!!!
