# Supplementary material for: A counseling program on nuisance bleeding improves quality of life in patients on dual antiplatelet therapy: A randomized controlled trial
Source: PLoS One. 2017 Aug 23;12(8):e0182124. doi: 10.1371/journal.pone.0182124 (PMC5568410; doi:10.1371/journal.pone.0182124)
Supplement: S3 File — (PDF) [file pone.0182124.s003.pdf]

SERVIZIO SANITARIO REGIONALE
EMILIA-ROMAGNA
Azienda Ospedaliero - Universitaria di Ferrara

UNIVERSITÀ DEGLI STUDI DI FERRARA
- EX LABORE FRUCTUS -

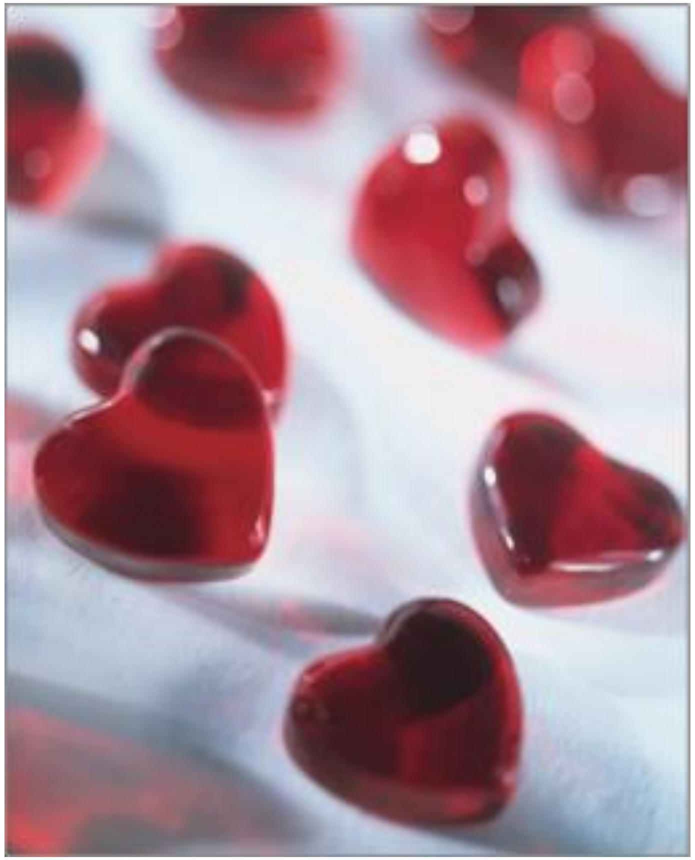

ANTIPLATELET DRUGS AND MINOR BLEEDINGS

Patient's guide

What are antiplatelet drugs?

Antiplatelet agents are drugs that can reverse the natural tendency of platelets to form blood clots. This mechanism is aimed at stopping bleeding in case of injury, but it is the cause of myocardial infarction when a clot formed because of a plaque rupture occludes the coronary artery. Thus, in some cases, clots formation needs to be prevented in order to avoid formation of thrombus in the vessels. This is particularly true after stent implantation, a medical device that is recognized by our immunologic defenses leading to activation of coagulation process.

How many antiplatelet drugs exist? What does dual antiplatelet therapy mean?

One of the most used and efficient antiplatelet drug is Aspirin (acetylsalicylic acid), which may be used in association with Clopidogrel (Plavix), Prasugrel (Efient) or Ticagrelor (Brilique) in dual antiplatelet therapy. These active substances are different in term of efficacy and adverse events (mainly in bleeding risk), but their action on platelets is analogous. As Aspirin and the other antiplatelet agents act on two different pathways, dual antiplatelet therapy maximizes the inhibitory effect on platelet activity.

How long do I have to stay on dual antiplatelet therapy?

Your cardiologist will give you specific information about dosage, modality and time of therapy according to your diagnosis (stable angina usually needs shorter therapy duration, unstable angina and acute myocardial infarction usually need longer therapy duration) and type of stent implanted (shorter therapy for bare metal stent and longer for drug eluting stent).

It is very important to meticulously follow the indications provided by your cardiologist, because a premature interruption of the therapy may lead to a new acute myocardial infarction!!! In case you need to undergo surgery or dental procedures it is mandatory to consult your cardiologist before modifying antiplatelet therapy.

REMEMBER: DO NOT INTERRUPT OR MODIFY DUAL ANTIPLATELET THERAPY ON YOUR OWN!

Can antiplatelet drugs cause bleeding?

Yes, they can. Because of their inhibiting effect on clots formation, you may experience longer bleeding in case of nosebleed or injury, and formation of larger bruises after small traumas. Some of those drugs may also decrease platelets and white cell count, therefore periodical blood count exam after the first weeks of therapy is strongly advised.

However, it is very important to highlight the benefits of such drugs with regards to ischemic protection, which outweighs by far the risk of bleeding. For these reasons, dual antiplatelet therapy must not be stopped because of minor bleeding, which can be easily managed. Below we detail the management of all possible bleedings occurring during dual antiplatelet therapy.

<u>What to do in the case of:</u>

Nosebleed (epistaxis)

Gently introduce a gauze soaked in Ugurol or Tranex in the bleeding nostril. Put some pressure pushing continuously the nose between two fingers for about 5-10 minutes, with your head slightly tilted backwards. After about half an hour, drop some other Ugurol or Tranex into the inserted gauze and when it is quite wet, remove it. If the bleeding does not stop, go to your general practitioner or to the closest emergency room. Epistaxis may be caused by high blood pressure, rupture of a mucosa varix, a sneeze during rhinitis or a cold, misuse of some medications.

Bleeding gums

Gums tend to bleed only when inflamed or injured. In order to reduce risk of gums bleeding some rules are needed, such as a correct use of the toothbrush which has to be of medium-bristled and with nylon bristle (avoid natural bristle brushes). Bleeding gums are a frequent event and usually not serious.

Small superficial injuries

Small superficial injuries are usually not serious: it is possible to stop the bleeding using a gauze soaked in Tranex or Ugurol and exercising a decisive and continued pressure on the wound for at least 10 minutes. If the bleeding persists, a tight bandage may be required as

well as advice from your general practitioner or the closest first-aid center.

Large bruising

Appearance of small or large bruises in every part of the body in the aftermath of experienced (even small) traumas are normal during antiplatelet therapy. If these are extended in size and appear spontaneously in the absence of any prior trauma, it is recommended to contact your general practitioner for advice.

Blood in sputum

Blood in sputum may come from gums, throat (for example during pharyngitis) and bronchus. Consult your general practitioner for further evaluations.

Conjunctival hemorrhage (broken blood vessel in the eye)

Cough, sneeze, traumas, even of small entity, or exposure to cold currents, can cause hemorrhagic extravasation at the level of the conjunctiva: the eye tends to become red and to fill with blood. Normally, such bleeding resolves on its own without further consequences. However, complete recovery may take up to 15 days. At this regard, regularly checking blood pressure may be useful. In the event of extended and protracted hemorrhagic episodes, disturbed vision discomfort due to the feeling of having a foreign body in your eye, pain to the eye-bulb, immediately contact your general practitioner.

Blood in urine (hematuria)

Red urine is never normal: it may be symptom of bladder or kidney diseases, as urinary tract infections or nephritis, which may present themselves even in the absence of any additional symptom (i.e. burning, pain, sense of discomfort in your lower abdomen) you are advised to contact your general practitioner and run relevant blood tests.

Blood in stool

If you have spotted few drops of blood in your stool and if these are bright in color, these are probably due to hemorrhoids. If these are dark in color or if your stool tend to the black color, contact your general practitioner. If you are experiencing consistent and continuous bleeding, immediately reach the closest emergency room.

Bloody vomit (hematemesis)

In case of bloody vomit immediately contact the emergency number.

If you need any further information please do not hesitate to contact us!

Study Staff:

Simone Biscaglia MD

Gianluca Campo MD

Matteo Tebaldi MD

Carlo Tumscitz MD

Rita Pavasini MD

Matteo Serenelli MD

Elisabetta Tonet MD

Giulia Bugani MD

Paolo Cimaglia MD

Francesco Gallo MD

Giosafat Spitaleri MD

Del Franco Anna Maria MD

Study Coordinators:

Veronica Lodolini BSc

Elisa Mosele BSc

Phone numbers: +39053223-7227; +39053223-6450

Email addresses:

emodinamica.ferrara@gmail.com

cardiologia@ospfe.it
