# Supplementary material for: A counseling program on nuisance bleeding improves quality of life in patients on dual antiplatelet therapy: A randomized controlled trial
Source: PLoS One. 2017 Aug 23;12(8):e0182124. doi: 10.1371/journal.pone.0182124 (PMC5568410; doi:10.1371/journal.pone.0182124)
Supplement: S4 File — (PDF) [file pone.0182124.s004.pdf]

SERVIZIO SANITARIO REGIONALE
EMILIA-ROMAGNA
Azienda Ospedaliero - Universitaria di Ferrara

UNIVERSITÀ DEGLI STUDI DI FERRARA
- EX LABORE FRUCTUS -

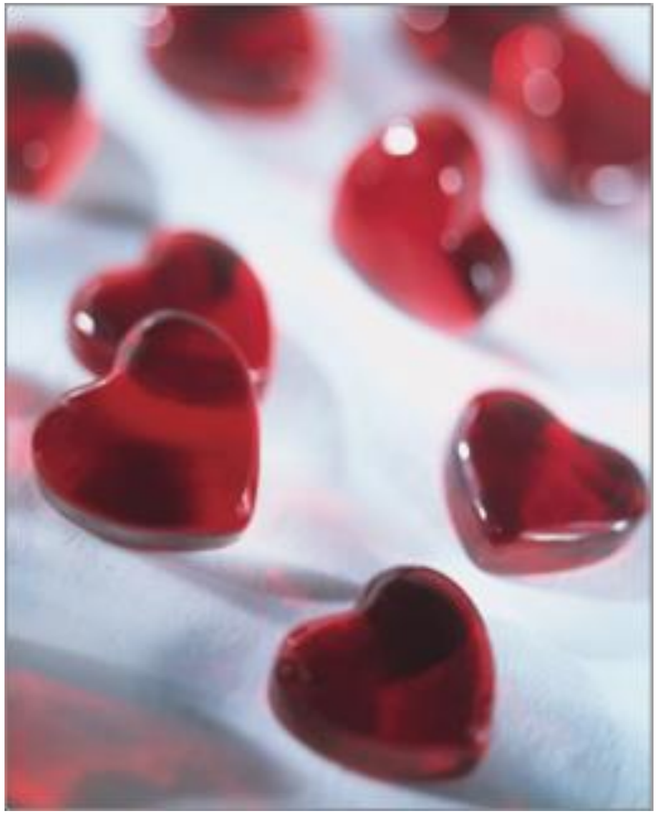

ANTIPLATELET DRUGS AND MINOR BLEEDINGS

General practitioner's disclosure

Introduction

In the setting of acute coronary syndrome, the availability of percutaneous coronary intervention significantly reduced mortality, re-infarction and re-hospitalisation. Despite recent improvements, coronary stenting still presents some drawbacks, such as stent thrombosis, an infrequent but life-threatening event. Dual anti-platelet therapy, consisting of Aspirin (ASA) and P2Y12 ADP receptor inhibitors is the first choice of treatment to reduce cardiovascular events and intra-stent thrombosis rate. Studies showed that dual antiplatelet therapy (DAPT) adoption significantly increases life expectancy, even for those patients subject only to medical therapy.

There are two major determinants of DAPT duration:

1. Patient's presentation: stable coronary artery disease (SCAD) vs. acute coronary syndrome (ACS);
2. Type of implanted stent: Bare Metal Stents (BMS) vs. Drug Eluting Stents (DES).

DAPT length

This information is relevant because DAPT length is usually tailored according these two characteristics as follows:

	SCAD	ACS
DES	6 months	12 months
BMS	1 months	12 months

Our usual strategy in most of the patients undergoing PCI with DES is to stop DAPT after 12 months, balancing ischemic and bleeding risk.

Major bleedings

The occurrence of a major bleeding in a patient on DAPT because of a recent ACS dramatically worsens his/her prognosis. Therefore, it is recommended to downgrade antiplatelet therapy to reduce risk of complication in patients experiencing a major bleeding. If the patient received a BMS at least 30 days before, therapy adjustment is relatively straightforward, and usually involves withdrawal of one antiplatelet drug. In patients implanted with second generation DES, DAPT should not be suspended before 3 (possibly 6) months of therapy. If a major bleeding will occur the patient will be usually hospitalized and a cardiologist will manage DAPT.

However, if you have any doubt dealing with a patient on DAPT with a recent major bleeding please feel free to contact us. We will be more than happy to help you in the management of DAPT.

In case of major bleeding it is possible to switch from DAPT to antiplatelet monotherapy, possibly not earlier than 30 days for BMS and 6 months for DES.

Minor/nuisance bleedings

Recent studies (1,2) have shown that even minor (nuisance) bleedings impact on patients' quality of life and DAPT adherence. In case of *nuisance* bleedings, it is mandatory to provide patients with complete and adequate information, preventing them from DAPT withdrawal. Patients should be reassured about the necessity to continue DAPT and about the clinical benefits deriving from DAPT. Only in cases of clinically relevant major bleedings or where serious corrective measures become necessary (for example uncontrolled hematuria), a shift to Clopidogrel in patients treated with Ticagrelor or Prasugrel may be considered. The use of low molecular weight Heparin is not a feasible alternative to antiplatelet drug as it acts through a different mechanism that does not reduce arterial thrombosis risk. Thus, never substitute an antiplatelet agent with heparin.

In case of minor (nuisance) bleeding it is pivotal to reassure patients about the necessity to continue DAPT. Never withdraw DAPT because of minor bleeding.

1. Amin AP et al. Nuisance bleeding with prolonged dual antiplatelet therapy after acute myocardial infarction and its impact on health status. J Am Coll Cardiol. 2013;61:2130-38.
2. Amin AP et al. Impact of Bleeding on Quality of Life in Patients on DAPT: Insights From TRANSLATE-ACS. J Am Coll Cardiol. 2016;67:59-65.

If you need any further information please do not hesitate to contact us!

Study Staff:

Simone Biscaglia MD

Gianluca Campo MD

Matteo Tebaldi MD

Carlo Tumscitz MD

Rita Pavasini MD

Matteo Serenelli MD

Elisabetta Tonet MD

Giulia Bugani MD

Paolo Cimaglia MD

Francesco Gallo MD

Giosafat Spitaleri MD

Del Franco Annamaria MD

Study Coordinators:

Veronica Lodolini BSc

Elisa Mosele BSc

Phone numbers: +390532237227; +390532236450

Email addresses:

emodinamica.ferrara@gmail.com

cardiologia@ospfe.it
