# Supplementary material for: A counseling program on nuisance bleeding improves quality of life in patients on dual antiplatelet therapy: A randomized controlled trial
Source: PLoS One. 2017 Aug 23;12(8):e0182124. doi: 10.1371/journal.pone.0182124 (PMC5568410; doi:10.1371/journal.pone.0182124)
Supplement: S5 File — (PDF) [file pone.0182124.s005.pdf]

UNIVERSITÀ DEGLI STUDI DI FERRARA
- EX LABORE FRUCTUS -

SERVIZIO SANITARIO REGIONALE
EMILIA-ROMAGNA
Azienda Ospedaliero - Universitaria di Ferrara

Impact of nuisance bleeding on quality of life

NAME:………
SURNAME: ………………………………………………………………………………………………………
DATE OF BIRTH: …………………………………………………………………………………………
ACTUAL ANTIPLATELET THERAPY:……………………………………………………………………
ANTIPLATELET THERAPY STARTING DATE (specify for each drug):………
……

From discharge:

		QUESTIONS:			
SECTION A	Evidence of nuisance bleeding	1	Have you ever experienced any nuisance bleeding?	NO	YES
		2	Have you ever had nose bleedings?	NO	YES
		3	Have you ever had gum bleedings?	NO	YES
		4	Have you ever noticed small (1–2 mm) red or purple spots on the skin?	NO	YES
		5	Have you ever noticed small bruising or hematomas on the skin not related to a trauma?	NO	YES
		6	Have you ever had any small conjunctival hemorrhage?	NO	YES
		7	Have you ever noticed spontaneous bleedings from privates?	NO	YES
		8	Have you ever noticed small rectal bleedings?	NO	YES
SECTION B	Nuisance bleeding management	Information request			
		9	Did you look for information regarding risks and benefits of dual antiplatelet therapy?	NO	YES
		10	If you answered yes to question number 9, did you call your general practitioner?	NO	YES
		11	If you answered yes to question number 9, did you ask to your friends/relatives?	NO	YES
		12	If you answered yes to question number 9, did you call your private cardiologist?	NO	YES
		13	If you answered yes to question number 9, did you look on the internet?	NO	YES
		14	Was your information request related to nuisance bleedings (see questions 1-8)	NO	YES
		15	If you answered yes to any question from 1 to 8, did you ask for a consult with your general practitioner?	NO	YES
		16	If you answered yes to any question from 1 to 8, did you go to the emergency room?	NO	YES
		17	In case of bleeding, have you been hospitalized?	NO	YES
		18	Did you withdraw antiplatelet therapy because of the bleeding?	NO	YES
		19	If yes, was it a temporary withdrawal?	NO	YES
		20	Who did recommend the drug withdrawal? …………………………………………………………………………		
		21	How long was the suspension? ………………………………………………………………………………………		
		22	Was the drug substituted?	NO	YES
		23	If yes, to which drug was the patient switched? ……………………………………………………………		
		24	Did you reschedule dental care because of the antiplatelet therapy?	NO	YES
		25	Did you reschedule eye care because of the antiplatelet therapy?	NO	YES
		26	Did you undergo dental care?	NO	YES
		27	Did you undergo dental care?	NO	YES
		28	If you answered yes to question 26 or 27, did you have to withdraw antiplatelet therapy?	NO	YES
		29	If yes, who prescribed the drug suspension?……………………………………………………………………………		
		30	Which drug did you suspend?……………………………………………………………		
		31	How long was the suspension? ……………………………………………………………		
		32	Was the drug substituted?	NO	YES
		33	If yes, to which drug were you switched to? …………………………………………………………………		
SECTION C	Bleeding impact on QoL EQ-5D	Pain/discomfort			
		34	I have no pain or discomfort	NO	YES
		35	I have moderate pain or discomfort	NO	YES
		36	I am in extreme pain or discomfort	NO	YES
		Anxiety/Depression			
		37	I am not anxious or depressed	NO	YES

		38	I am moderately anxious or depressed	NO	YES
		39	I am extremely anxious or depressed	NO	YES
		Mobility			
		40	I have no problems in walking about	NO	YES
		41	I have some problems in walking about	NO	YES
		42	I am confined to bed	NO	YES
		Self-Care			
		43	I have no problems with self-care	NO	YES
		44	I have some problems washing or dressing myself	NO	YES
		45	I am unable to wash or dress myself	NO	YES
		Usual Activities (e.g. work, study, housework, family or leisure activities)			
		46	I have no problems with performing my usual activities	NO	YES
		47	I have some problems with performing my usual activities	NO	YES
		48	I am unable to perform my usual activities	NO	YES
		49	Do you think that the presence of small bleedings influenced/limited your sexual activity?	NO	YES
		50	Do you think that the presence of small bleedings influenced/limited your work activity?	NO	YES
		51	Do you think that the presence of small bleedings influenced/limited in your interpersonal relationships?	NO	YES
