# Supplementary material for: A counseling program on nuisance bleeding improves quality of life in patients on dual antiplatelet therapy: A randomized controlled trial
Source: PLoS One. 2017 Aug 23;12(8):e0182124. doi: 10.1371/journal.pone.0182124 (PMC5568410; doi:10.1371/journal.pone.0182124)
Supplement: S6 File — (PDF) [file pone.0182124.s006.pdf]

Predischarge Checklist

The discharging physician must control his/her prescriptions and deal with all the following items before patient's discharge (check each box).

EKG acquisition ☐

DAPT prescription ☐

Explanation of DAPT risks and benefits ☐

Follow-up visit scheduling, ☐

Explanation of Heart Failure management (with scheduled follow-up visit to Heart Failure center if indicated) ☐

Explanation of Diabetes management (with scheduled follow-up visit to Diabetes center if indicated) ☐

Explanation of lifestyle adjustments ☐

Discharging Physician Signature...
