# Supplementary material for: A counseling program on nuisance bleeding improves quality of life in patients on dual antiplatelet therapy: A randomized controlled trial
Source: PLoS One. 2017 Aug 23;12(8):e0182124. doi: 10.1371/journal.pone.0182124 (PMC5568410; doi:10.1371/journal.pone.0182124)
Supplement: S7 File — (DOCX) [file pone.0182124.s007.docx]

**Title.**

Predischarge Bundle for Patients in Dual Antiplatelet Therapy to Minimize the Negative

Impact of Nuisance Bleedings on Quality of Life: a Randomized Controlled Trial.

**Unit involved:**

- Cardiology Unit, Azienda Ospedaliero-Universitaria S.Anna di Ferrara

**Research responsible:**

Gianluca Campo, Cardiology Unit, Azienda Ospedaliero-Universitaria S.Anna di Ferrara, MD

**INTRODUCTION**

Antiplatelet therapy is the milestone for the pharmacologic treatment in patients with ischemic heart disease [1]. In the last years, attention has grown towards bleedings incidence due to antiplatelet therapy and towards their clinical impact. Some randomized studies included major bleedings as safety and efficacy endpoint [2].

It is well known that guidelines recommend stratification of ischemic and bleeding risk in patients with ischemic heart disease [3]. This stratification is of paramount importance because there is a strict interaction between potency of antithrombotic therapy and bleeding incidence. Thus, the ability of the clinician is to fall in the ideal therapeutic window in which the risk of ischemic and bleeding events is minimal.

Bleeding can cause shock, anemia, blood transfusions which are associated in the activation of inflammatory and thrombosis mechanisms. Yet, even the reduction or the withdrawal of antithrombotic drugs can cause reoccurrence of ischemic events. All these events translate in higher mortality in the long-term.

Different factors are responsible for the bleeding risk in the ischemic heart disease setting: patient-related (i.e.: body weight, renal function, comorbidities), drug-related (drug potency, therapeutic window, therapy duration), quality of treatment related (number of antithrombotic drug used, pharmacological associations, eventual invasive strategy, vascular access, underutilization of gastro-protectors, therapy adherence, complications management) [4,5].

The vast majority of clinical trials evaluated the effect of dual antiplatelet therapy on major bleedings, defined with the TIMI classification (Thrombolysis In Myocardial Infarction) and GUSTO (Global Use of Strategies to Open Coronary Arteries) [6,7,8,9]. The incidence of minor bleeding events is mostly unknown. At the same time, so-called nuisance bleedings are frequent in everyday clinical practice in patients on dual antiplatelet therapy and they are often associated with early drug withdrawal [10] and with adverse cardiac events.

Thus, a new bleeding classification has been proposed, taking into account the wide-spectrum of bleeding complications, including superficial/minor bleedings [11]. The BARC classification (Bleeding Academic Research Consortium) defines as type 1 those bleedings not actionable and not causing unscheduled visits or treatment. In addition, antiplatelet drugs self-discontinuation without physician consult are included [11].

At the present time, there are few studies, single-center and with low bleeding-risk patients, analyzing the long-term incidence and clinical implication of nuisance bleeding in patients after percutaneous coronary intervention [10,12]. Another relevant aspect is the impact of nuisance bleeding on patient’s quality of life [13].

**REFERENCES.**

1. Levine GN, Ali MN, Schafer AI. Antithrombotic therapy in patients with acute coronary syndromes. Arch Intern Med 2001;161:937-48.
2. Dauerman HL. Percutaneous coronary intervention pharmacology: from a triangle to a square. J Am Coll Cardiol 2008;51:698-700.
3. Steg PG, James SK, Atar D, et al.; Task Force on the management of ST-segment elevation acute myocardial infarction of the European Society of Cardiology (ESC). ESC Guidelines for the management of acute myocardial infarction in patients presenting with ST-segment elevation. Eur Heart J 2012;33:2569-619.
4. Lawler PR, Filion KB, Dourian T, Atallah R, Garfinkle M, Eisenberg MJ. Anemia and mortality in acute coronary syndromes: a systematic review and meta-analysis. Am Heart J 2013;165:143-53.
5. Willis P, Voeltz MD. Anemia, hemorrhage, and transfusion in percutaneous coronary intervention, acute coronary syndromes, and ST-segment elevation myocardial infarction. Am J Cardiol 2009;104(5 Suppl):34C-8C.
6. Yusuf S, Zhao F, Mehta SR, Chrolavicius S, Tognoni G, Fox KK; Clopidogrel in Unstable Angina to Prevent Recurrent Events Trial Investigators. Effects of clopidogrel in addition to aspirin in patients with acute coronary syndromes without ST-segment elevation. N Engl J Med 2001;345:494 –502.
7. Steinhubl SR, Berger PB, Mann JT III, Fry ET, DeLago A, Wilmer C, Topol EJ; CREDO Investigators. Early and sustained dual oral antiplatelet therapy following percutaneous coronary intervention: a randomized controlled trial. JAMA 2002;288:2411–2420.
8. Diener HC, Bogousslavsky J, Brass LM, Cimminiello C, Csiba L, Kaste M, Leys D, Matias-Guiu J, Rupprecht HJ; MATCH Investigators. Aspirin and clopidogrel compared with clopidogrel alone after recent ischaemic stroke or transient ischaemic attack in high-risk patients (MATCH): randomised, double-blind, placebo-controlled trial. Lancet 2004;364:331–337.
9. Bhatt DL, Fox KA, Hacke W, Berger PB, Black HR, Boden WE, Cacoub P, Cohen EA, Creager MA, Easton JD, Flather MD, Haffner SM, Hamm CW, Hankey GJ, Johnston SC, Mak KH, Mas JL, Montalescot G, Pearson TA, Steg PG, Steinhubl SR, Weber MA, Brennan DM, Fabry-Ribaudo L, Booth J, Topol EJ; CHARISMA Investigators.)
10. Roy P, Bonello L, Torguson R, de Labriolle A, Lemesle G, Slottow

TL, Steinberg DH, Kaneshige K, Xue Z, Satler LF, Kent KM, Suddath WO, Pichard AD, Lindsay J, Waksman R. Impact of “nuisance” bleeding on clopidogrel compliance in patients undergoing intracoronary drug-eluting stent implantation. Am J Cardiol 2008;102:1614–1617.

1. Roxana Mehran, MD; Sunil V. Rao, MD; Deepak L. Bhatt, MD, MPH; C. Michael Gibson, MS, MD; Adriano Caixeta, MD, PhD; John Eikelboom, MD, MBBS; Sanjay Kaul, MD; Stephen D. Wiviott, MD; Venu Menon, MD; Eugenia Nikolsky, MD, PhD; Victor Serebruany, MD, PhD; Marco Valgimigli, MD, PhD; Pascal Vranckx, MD; David Taggart, MD, PhD; Joseph F. Sabik, MD; Donald E. Cutlip, MD; Mitchell W. Krucoff, MD; E. Magnus Ohman, MD; Philippe Gabriel Steg, MD; Harvey White, MB, ChB, DScStandardized Bleeding Definitions for Cardiovascular Clinical Trials A Consensus Report From the Bleeding Academic Research ConsortiumCirculation. 2011;123:2736-2747.)
2. Ben-Dor I, Torguson R, Scheinowitz M, et al. Incidence, correlates, and clinical impact of nuisance bleeding after antiplatelet therapy for patients with drug-eluting stents. Am Heart J 2010;159:871–5.
3. Amit P. Amin, MD, MSC, Alok Bachuwar, MD, Kimberly J. Reid, MS, Adnan K. Chhatriwalla, MD, Adam C. Salisbury, MD, MSC, Robert W. Yeh, MD, MSC, Mikhail Kosiborod, MD, Tracy Y. Wang, MD, MHS, Karen P. Alexander, MD, Kensey Gosch, MS, David J. Cohen, MD, MSC, John A. Spertus, MD, MPH, Richard G. Bach, MD. Nuisance Bleeding With Prolonged Dual Antiplatelet Therapy After Acute Myocardial Infarction and its Impact on Health Status. JACC Vol. 61, No. 21, 2013 May 28, 2013:2130–8.**Study Design.**

This is a prospective study.

We will enroll consecutive patients hospitalized in our Cardiology Unit of Ferrara because of ischemic heart disease undergoing percutaneous coronary intervention and second generation drug eluting stent implantation in which dual antiplatelet therapy is indicated for at least 6 months. The actual enrollment will happen before the discharge from the Cardiology Unit.

Patients will be randomized in a 1:1 fashion to:

- Standard procedure: delivery of the discharge letter and recommendations regarding lifestyle and drug prescriptions as per routine process in our unit. In our everyday clinical practice, the discharge letter is given to the patient from the treating Cardiologist of the ward. In this occasion, the physician briefly resumes what has happened during the hospitalization and he/she focuses on the pharmacological therapy with clear indications about duration and posology of the therapies. During this talk (usually lasting 15 minutes), the physician tries to explain the reason and the effect of every prescription. Finally, he/she recommends to adopt a proper lifestyle in order to reduce the cardiovascular risk burden (physical activity, hypolipidic and hyposodic diet, smoking cessation).
- Experimental procdure. Patients in the experimental arm will receive the following (in addition to the standard procedure):

1. a 15-minutes meeting with a member of the research team 24 hours prior discharge. During this visit, a core set of DAPT risks were addressed. DAPT advantages and side effects were described. The importance of compliance and the correct management of side effects (especially BARC 1 bleedings) were discussed;
2. a 15-minutes meeting with a member of the research team with a patient’s next of kin addressing the same issues: DAPT advantages and side effects, importance of compliance, side effect management;
3. a brochure describing DAPT advantages, side effects, and management;
4. a brochure for the patient’s general practitioner aimed at presenting DAPT rationale and management. At the same time, study investigators directly contacted the patient’s general practitioner by phone and/or by email describing and explaining the same topics and the clinical picture of his/her patient (see eMethods);
5. a phone number to further discuss potential side effects of DAPT and to contact before any decision about DAPT withdrawal. The phone number was active from Monday to Friday from 9 am to 12 am. A study coordinator received the phone calls and, if deemed necessary, contacted one of the study group physicians to deal with patients’ requests;
6. two phone calls per month by the study coordinator to assess DAPT compliance and potential BARC 1 bleedings.

**MAIN DIFFERENCES BETWEEN STANDARD PRACTICE AND EXPERIMENTAL PROCEDURE.**

The main differences between the experimental arm and the control arm are:

- the discharging physician will be joined by a study member in order to reinforce the message regarding importance of dual antiplatelet therapy in patients treated with percutaneous coronary intervention;
- during the talk there will be time to explain in detail antiplatelet therapy and the importance of therapy adherence;
- the talk will be addressed not only to the patient but it will also be repeated to a patient’s next of kin who daily interacts with the patient;
- the patient and his/her relatives will receive a brochure with all information that can be read at home
- the counseling will be focused not only on dual antiplatelet therapy benefits but also on potential side effects. These are usually never discussed during the discharge process. The aim is exactly to inform patients and relatives on the possible side effects, but most importantly to discriminate between important and not important side effects. Moreover, the physician will explain in detail to the patient how to handle eventual side effects, which are the red flags and when it is necessary to contact emergency service or the general practitioner;
- the counseling activity will be mainly dedicated to the description of minor bleedings and on how to distinguish between those important from a prognostic point of view. Moreover, it will be clarified that therapy withdrawal is not the solution of this problems and carries out a significant risk of reinfarction or sudden death.

During the 1-month follow-up visit, every patient will receive a questionnaire about nuisance bleedings and their impact on quality of life and drug adherence. Moreover, all patients will receive a questionnaire regarding other potential side effects (i.e.: dyspnea related to ticagrelor).

**Materials and methods**

Before discharge, patients will be informed regarding the aim and modalities of the study and, if willing to participate, after reading the rationale and the informative materials, will sign the informed consent. A copy of the informative materials will be left to the patient. All patients will be consecutively enrolled. Randomization will be executed via sealed envelopes.

Patients randomized to the experimental arm will receive information regarding antiplatelet therapy and nuisance bleeding as well as a brochure with the same information. In the next page, you can find the patients’ brochure.

Patients’ brochure

**Study flow chart.**

Hospitalization for ischemic heart disease

PCI with DES implantation and indication to at least 6 months of DAPT

Discharge letter delivery (standard procedure)

Counseling and brochures

(experimental procedure)

1-month Follow-up: cardiologic evaluation, questionnaire regarding bleedings and side effects, QoL, evaluation of every potential cardiac and non cardiac side effect

**PRIMARY OBJECTIVE OF THE STUDY.**

6-months Follow: questionnaire regarding bleedings and side effects, QoL, evaluation of every potential side cardiac and non cardiac side effect

- Evaluate if the experimental procedure is able to impact on quality of life at 1 month, through the EQ-5D VAS instrument, in patients treated with PCI and on dual antiplatelet therapy.

**SECONDARY OBJECTIVES OF THE STUDY.**

- Evaluate if the experimental procedure is able to impact on quality of life at 6 months, through the EQ-5D VAS instrument, in patients treated with PCI and on dual antiplatelet therapy.
- Evaluate if the experimental procedure is able to impact on quality of life at 1 months, through the EQ-5D instrument, in patients treated with PCI and on dual antiplatelet therapy.
- Evaluate if the experimental procedure is able to impact on quality of life at 6 months, through the EQ-5D instrument, in patients treated with PCI and on dual antiplatelet therapy.
- Evaluate if the experimental procedure is able to reduce the number of accesses to emergency room and/or general practitioner.
- Evaluate if the experimental procedure is able to reduce the number of dual antiplatelet therapy withdrawal (temporary or permanent).
- Evaluate the incidence of nuisance bleedings in prespecified subgroups (ie: diabetics, COPD, CKD, female).
- Evaluate the incidence of DAPT side effects in prespecified subgroups (ie: diabetics, COPD, CKD, female) and in relation to the specific drug (clopidogrel vs. ticagrelor vs. prasugrel).
- Evaluate the incidence of non cardiac dyspnea in prespecified subgroups (ie: diabetics, COPD, CKD, female) and in relation to the specific drug (clopidogrel vs. ticagrelor vs. prasugrel).

**INCLUSION CRITERIA**

In order to include a patient in the study all the following criteria must be respected:

- - Sign of the informed consent.
  - Hospital admission for ischemic heart disease (see definition).
  - PCI with at least 1 stent implantation.
  - Indication to at least 6 months of DAPT.

**EXCLUSION CRITERIA**

- - Chronic therapy with oral anticoagulant.
  - Major bleeding in the last 30 days.
  - Planned surgery, included CABG within 6 months.

**DEFINITION OF ISCHEMIC HEART DISEASE.**

The term “ischemic heart disease” includes a series of different clinical manifestations, which have in common the development of myocardial ischemia, a suffering or damage to the myocardial cells due to a mismatch between oxygen delivery and metabolic request.

Coronary atherosclerosis is by far the most frequent cause of ischemic cardiopathy, which can be also caused by coronary spasm, microcirculation imbalance or extra coronary causes.

Ischemic cardiopathy can occur as:

- - Chronic ischemic cardiopathy: stable angina; microvascular angina; variant angina; dilatative post ischemic cardiopathy.
  - Acute coronary syndrome: ST elevated acute myocardial infarction; non ST elevated acute myocardial infarction; unstable angina.

**DEFINITION AND EVALUATION METHODS OF QUALITY OF LIFE.**

EuroQoL is a standardized instrument to evaluate the health status of people and their quality of life. On its basis, it is possible to evaluate the level of sanitary assistance, of a technique or a technology. EuroQoL is based on a simple and short questionnaire divided into two sections: in the first there are 5 items regarding the health current status of the patient, each one with the possibility to choose the level of gravity (no, moderately, extremely). The items are: 1. Mobility, 2. Self-care, 3. Usual activities, 4. Pain/discomfort, 5. Anxiety/depression.

The second section includes a visual analog scale (VAS) through which patients estimate their overall health status on a 20-cm VAS with the endpoints being “best imaginable health state” (score=100) and “worst imaginable health state” (score=0) (14). The EQ-5D VAS is a quantitative measure of patients’ perceived health, a patient-centered health outcome. [1]

For the present study, we decided to use an adapted and validated form of the EQ-5D questionnarie (see below). The first part has been ideated to investigate nuisance bleedings and the actions caused by nuisance bleedings. The second part is actually the EQ-5D with the same 5 items and with the EQ-5D VAS.

Patients’ questionnaire


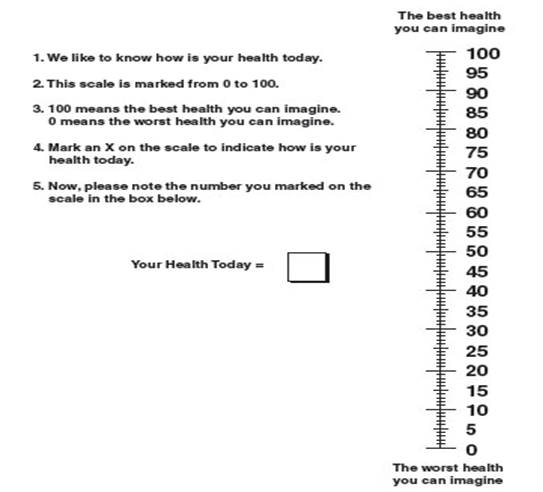


**DEFINITION OF TICAGRELOR-RELATED DYSPNEA**

Ticagrelor is a antiplatelet agent with a structure similar to the one of adenosine. It works inhibiting the platelet receptor ADP P2Y12 with consequent inhibition of platelet aggregation. Thus, its mechanism of action and clinical structure suggest that the profile of adverse events is similar to the one of adenosine that can cause dyspnea (also with bronchospasm), vasodilatory effects and atrioventricular conduction blocks. Incidence of dyspnea in patients treated with ticagrelor is common, usually mild and transitory. It usually appears within the first week of treatment and can last even few minutes or also can last for weeks. However, it does not seem to be related to any pulmonary or cardiac sequela.

**MONITORED VARIABLES**

|  | Discharge | 1 month | 6 months |
| --- | --- | --- | --- |
| Mini routine | x | x |  |
| ECG | x | x |  |
| Objective exam | x | x |  |
| Cardiovascular Anamnesis | x |  |  |
| Current cardiac therapy | x | x | x |
| Randomization | x |  |  |
| Counselling and brochure delivery | x | x |  |
| EQ-5D and EQ-5D VAS | x | x | x |
| Adverse events | x | x | x |

**MONITORED CLINCICAL EVENTS DURING FOLLOW-UP AND THEIR DEFINITIONS.**

- Death
- Myocardial infarction
- Stent thrombosis
- Bleedings defined with BARC classification
- Major or minor surgery
- Dental surgery
- Eye surgery
- Contacts or visits to the general practitioner
- Emergency room access
- Antiplatelet therapy withdrawal (temporary or permanent)

**DEFINITION OF MYOCARDIAL INFARCTION (ESC GUIDELINES 2012)**

The term acute myocardial infarction is used in case of evidence of myocardial necrosis in a clinical setting of acute myocardial injury. Particularly, a typical rise and fall in troponin is needed (troponin should exceed the 99^th^ percentile of the upper reference limit) in presence of at least one of the following conditions:

- - Ischemia symptoms,
  - De novo modifications of ST segment or T waves, new left bundle block, new pathological Q waves at the ECG.
  - Evidence of loss of viable myocardium at imaging or new defects of cinetic regions,
  - Identification of intracoronary thrombus at coronary artery angiography.

**DEFINITION OF STENT THROMBOSIS**

In accordarce with the consensus document and with all randomized clinical trials, stent thrombosis will be classified as suggested by the Academic Research Consortium (ARC). Below, we report the ARC classification.


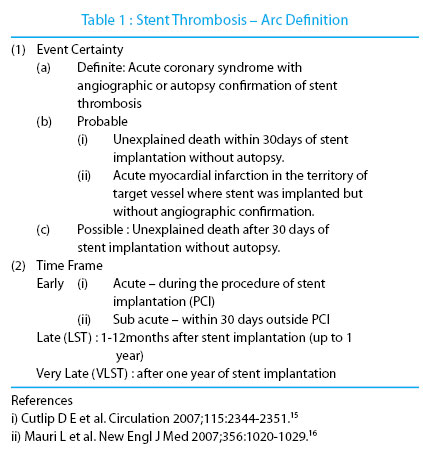


**DEFINITION OF BARC CLASSIFICATION**

For emorragic complications, we will apply the BARC (Bleeding Academic Research Consortium) classification. Below, we report the BARC classification.


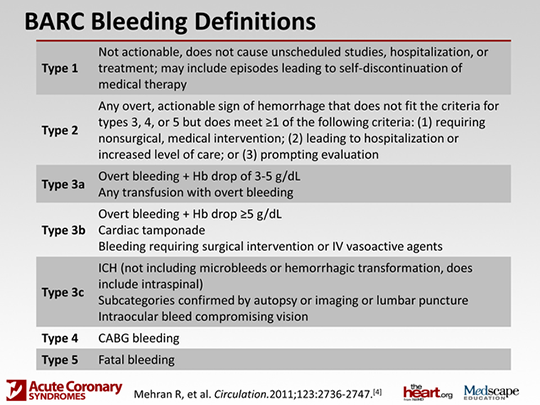


**SAMPLE SIZE**

For the sample size calculation, we will use as reference the work by Amin et al. [2]

The primary endpoint will be calculated on the VAS.

From the published data [2] and from our preliminary evaluations we can estimate that, at one month from the procedure, patients on DAPT will have a medium VAS of 75±19. We hypothesize that our counselling intervention will increase VAS value by 8%. Thus, it will be necessary to enroll at least 211 patients per group (α=0.05 and β=0.15, statistical potency 90%). The sample size has been inflated to 224 patients per group for possible dropout, lost to follow-up and consent withdrawal.

**Statistical plan**

All statistical analyses will be independently performed by the “Clinical and Epidemiological Research Center of the University of Ferrara”. Continuous variables with normal distribution will be expressed as medium value ± standard deviation. Continuous variables with non normal distribution will be expressed as medium value and interquartile range. Normality will be tested with Kolmogorov–Smirnov test or with the visual estimation of the Q-Q Plot. Categorical variables will be expressed as number and percentage (%). The comparison between variables will be executed with the χ2 test (Yates correction) if categorical, with the Student test or Satterthwaite test or with 1-way ANOVA if continuous with normal distribution, with Kruskal–Wallis test if continuous with non-normal distribution. We will describe the survival free from adverse events through the Kaplan-Meier method and the differences between the two groups will be estimated and calculated with the log-rank test. Adjusted estimate for all potential confounding factors will be obtained with Cox proportional hazard models, including all positive variables at the univariate analysis or clinically significative. A value of p<0.05 will be considered as significant. When appropriated, confidence intervals at 95% will be calculated. Alla analyses will be performed with STATISTICA 8 or STATA 10.

**REFERENCES.**

1. R. Rabin, F. De Charro “EQ-5D: A Measure of Health Status from the EuroQol Group”. The Finnish Medical Society Duodecim, Ann. Med. 2001; 33:337-343.
2. Amit P. Amin, MD, MSC, Alok Bachuwar, MD, Kimberly J. Reid, MS, Adnan K. Chhatriwalla, MD, Adam C. Salisbury, MD, MSC, Robert W. Yeh, MD, MSC, Mikhail Kosiborod, MD, Tracy Y. Wang, MD, MHS, Karen P. Alexander, MD, Kensey Gosch, MS, David J. Cohen, MD, MSC, John A. Spertus, MD, MPH, Richard G. Bach, MD. Nuisance Bleeding With Prolonged Dual Antiplatelet Therapy After Acute Myocardial Infarction and its Impact on Health Status. JACC Vol. 61, No. 21, 2013 May 28, 2013:2130–8.
