# Supplementary material for: A counseling program on nuisance bleeding improves quality of life in patients on dual antiplatelet therapy: A randomized controlled trial
Source: PLoS One. 2017 Aug 23;12(8):e0182124. doi: 10.1371/journal.pone.0182124 (PMC5568410; doi:10.1371/journal.pone.0182124)
Supplement: S8 File — (DOCX) [file pone.0182124.s008.docx]

**Titolo.**

Counselling prima della dimissione ospedaliera in pazienti trattati con duplice terapia antiaggregante per minimizzare l’impatto sulla qualità di vita dei sanguinamenti “nuisance”: studio prospettico randomizzato.

**Unità operative coinvolte:**

- U.O. di Cardiologia, Azienda Ospedaliero-Universitaria S.Anna di Ferrara

**Responsabile della ricerca:**

dott. Gianluca Campo, U.O. di Cardiologia, AOU di Ferrara

**INTRODUZIONE**

La terapia antiaggregante rappresenta il cardine del trattamento farmacologico dei pazienti affetti da cardiopatia ischemica [1]. Negli ultimi anni è stata sempre più posta l’attenzione all’incidenza dei sanguinamenti legati alle terapie antitrombotiche ed al loro relativo impatto clinico; alcuni studi randomizzati hanno infatti incluso le emorragie maggiori nella valutazione dell’endpoint di sicurezza e di efficacia [2].

E’ ben noto che le linee guida raccomandano il ricorso precoce alla stratificazione del rischio ischemico ed emorragico nei pazienti con cardiopatia ischemica [3]. Tale stratificazione risulta di fondamentale importanza poiché esiste una stretta correlazione tra l’aggressività della terapia antitrombotica e l’incidenza di emorragie, per cui l’abilità del clinico dovrebbe essere quella di collocarsi in una finestra terapeutica ideale nella quale è minimizzato il rischio di potenziali eventi ischemici ed emorragici.

L’emorragia può determinare shock, anemia, necessità di trasfusioni che si associano ad attivazione dei meccanismi di infiammazione e trombosi, ma molto spesso anche la riduzione o la sospensione dei trattamenti antitrombotici raccomandati può causare recidiva di eventi ischemici: tutto ciò si può tradurre in un aumento di mortalità a lungo termine. I fattori che condizionano il rischio emorragico nel setting della cardiopatia ischemica sono legati al paziente (es. peso corporeo, funzione renale, comorbilità), al farmaco (potenza del farmaco, finestra terapeutica, durata della terapia) ed alla qualità del trattamento (numero di farmaci antitrombotici utilizzati, associazioni farmacologiche, eventuale strategia invasiva, accesso vascolare, mancato uso di farmaci gastroprotettivi, aderenza alla terapia, e gestione delle complicanze)[4,5].

La maggior parte dei trial clinici ha valutato gli effetti di una duplice terapia antiaggregante sui sanguinamenti maggiori, definiti tramite la classificazione TIMI (Thrombolysis In Myocardial Infarction) e GUSTO (Global Use of Strategies to Open Coronary Arteries) [6,7,8,9]. L’incidenza di eventi emorragici minori è ancora oggi in gran parte sconosciuta. Di fatto, i cosiddetti sanguinamenti “nuisance” sono di comune riscontro nella pratica clinica in pazienti in duplice terapia antiaggregante e sono spesso associati a precoce sospensione della terapia [10] e quindi potenzialmente associati ad eventi avversi cardiaci.

È stata quindi messa a punto una nuova classificazione dei sanguinamenti, che comprende l’intero spettro di complicazioni emorragiche, inclusi i sanguinamenti superficiali o minori [11]. La classificazione BARC (Bleeding Academic Research Consortium) infatti definisce come tipo 1 quei sanguinamenti che non richiedono un intervento medico, una nuova ospedalizzazione o una visita aggiuntiva rispetto a quelle previste nel follow-up del paziente; sono inoltre inclusi i casi di auto-sospensione della terapia senza consulto medico specialistico [11].

Attualmente in letteratura esistono pochi studi, per lo più monocentrici e con casistiche di pazienti a basso rischio emorragico dopo angioplastica coronarica percutanea (PCI) [10,12] che analizzino l'incidenza a lungo termine e le implicazioni cliniche dei sanguinamenti “nuisance”. Un aspetto rilevante emerso è la correlazione tra la presenza di sanguinamenti “nuisance” ed il peggioramento della qualità di vita del paziente [13].**REFERENZE BIBLIOGRAFICHE RELATIVE ALL’INTRODUZIONE.**

1. Levine GN, Ali MN, Schafer AI. Antithrombotic therapy in patients with acute coronary syndromes. Arch Intern Med 2001;161:937-48.
2. Dauerman HL. Percutaneous coronary intervention pharmacology: from a triangle to a square. J Am Coll Cardiol 2008;51:698-700.
3. Steg PG, James SK, Atar D, et al.; Task Force on the management of ST-segment elevation acute myocardial infarction of the European Society of Cardiology (ESC). ESC Guidelines for the management of acute myocardial infarction in patients presenting with ST-segment elevation. Eur Heart J 2012;33:2569-619.
4. Lawler PR, Filion KB, Dourian T, Atallah R, Garfinkle M, Eisenberg MJ. Anemia and mortality in acute coronary syndromes: a systematic review and meta-analysis. Am Heart J 2013;165:143-53.
5. Willis P, Voeltz MD. Anemia, hemorrhage, and transfusion in percutaneous coronary intervention, acute coronary syndromes, and ST-segment elevation myocardial infarction. Am J Cardiol 2009;104(5 Suppl):34C-8C.
6. Yusuf S, Zhao F, Mehta SR, Chrolavicius S, Tognoni G, Fox KK; Clopidogrel in Unstable Angina to Prevent Recurrent Events Trial Investigators. Effects of clopidogrel in addition to aspirin in patients with acute coronary syndromes without ST-segment elevation. N Engl J Med 2001;345:494 –502.
7. Steinhubl SR, Berger PB, Mann JT III, Fry ET, DeLago A, Wilmer C, Topol EJ; CREDO Investigators. Early and sustained dual oral antiplatelet therapy following percutaneous coronary intervention: a randomized controlled trial. JAMA 2002;288:2411–2420.
8. Diener HC, Bogousslavsky J, Brass LM, Cimminiello C, Csiba L, Kaste M, Leys D, Matias-Guiu J, Rupprecht HJ; MATCH Investigators. Aspirin and clopidogrel compared with clopidogrel alone after recent ischaemic stroke or transient ischaemic attack in high-risk patients (MATCH): randomised, double-blind, placebo-controlled trial. Lancet 2004;364:331–337.
9. Bhatt DL, Fox KA, Hacke W, Berger PB, Black HR, Boden WE, Cacoub P, Cohen EA, Creager MA, Easton JD, Flather MD, Haffner SM, Hamm CW, Hankey GJ, Johnston SC, Mak KH, Mas JL, Montalescot G, Pearson TA, Steg PG, Steinhubl SR, Weber MA, Brennan DM, Fabry-Ribaudo L, Booth J, Topol EJ; CHARISMA Investigators.)
10. Roy P, Bonello L, Torguson R, de Labriolle A, Lemesle G, Slottow

TL, Steinberg DH, Kaneshige K, Xue Z, Satler LF, Kent KM, Suddath WO, Pichard AD, Lindsay J, Waksman R. Impact of “nuisance” bleeding on clopidogrel compliance in patients undergoing intracoronary drug-eluting stent implantation. Am J Cardiol 2008;102:1614–1617.

1. Roxana Mehran, MD; Sunil V. Rao, MD; Deepak L. Bhatt, MD, MPH; C. Michael Gibson, MS, MD; Adriano Caixeta, MD, PhD; John Eikelboom, MD, MBBS; Sanjay Kaul, MD; Stephen D. Wiviott, MD; Venu Menon, MD; Eugenia Nikolsky, MD, PhD; Victor Serebruany, MD, PhD; Marco Valgimigli, MD, PhD; Pascal Vranckx, MD; David Taggart, MD, PhD; Joseph F. Sabik, MD; Donald E. Cutlip, MD; Mitchell W. Krucoff, MD; E. Magnus Ohman, MD; Philippe Gabriel Steg, MD; Harvey White, MB, ChB, DScStandardized Bleeding Definitions for Cardiovascular Clinical Trials A Consensus Report From the Bleeding Academic Research ConsortiumCirculation. 2011;123:2736-2747.)
2. Ben-Dor I, Torguson R, Scheinowitz M, et al. Incidence, correlates, and clinical impact of nuisance bleeding after antiplatelet therapy for patients with drug-eluting stents. Am Heart J 2010;159:871–5.
3. Amit P. Amin, MD, MSC, Alok Bachuwar, MD, Kimberly J. Reid, MS, Adnan K. Chhatriwalla, MD, Adam C. Salisbury, MD, MSC, Robert W. Yeh, MD, MSC, Mikhail Kosiborod, MD, Tracy Y. Wang, MD, MHS, Karen P. Alexander, MD, Kensey Gosch, MS, David J. Cohen, MD, MSC, John A. Spertus, MD, MPH, Richard G. Bach, MD. Nuisance Bleeding With Prolonged Dual Antiplatelet Therapy After Acute Myocardial Infarction and its Impact on Health Status. JACC Vol. 61, No. 21, 2013 May 28, 2013:2130–8.**Disegno dello studio.**

Si tratta di uno studio prospettico.

Lo studio prevede l’arruolamento di pazienti consecutivi ricoverati presso l’U.O. di Cardiologia dell’AOU di Ferrara per cardiopatia ischemica, trattati con angioplastica coronarica e impianto di almeno uno stent in cui è stata posta indicazione a duplice terapia antiaggregante per almeno 6 mesi. L’arruolamento effettivo del paziente avverrà prima della dimissione dalla U.O. di Cardiologia.

Lo studio prevede per tutti i pazienti arruolati una randomizzazione 1:1 tra due gruppi:

- Consegna della lettera di dimissione e raccomandazione in merito allo stile di vita e alla prescrizione farmacologica come da normale routine in atto presso l’U.O. di Cardiologia (procedura standard). Nella nostra pratica clinica quotidiana la lettera di dimissione viene consegnata al paziente dal medico di reparto. La consegna della lettera di dimissione si accompagna da una breve sintesi di quello che si è fatto nel ricovero, ma soprattutto si concentra sulla terapia farmacologica che è stata impostata e come e per quanto deve essere assunta. In questo colloquio (di solito di circa 15 minuti) si cerca di spiegare perché ogni farmaco è stato prescritto e quale effetto si spera di ottenere con esso. Infine si raccomanda sempre di adottare uno stile di vita per minimizzare il rischio cardiovascolare (attività fisica, dieta iposodica e ipolipidica, cessazione del fumo).
- Alla procedura standard si abbineranno 5 interventi di counselling (procedura sperimentale):
  1. colloquio di almeno 15 minuti 24 ore prima della dimissione, tra medico dello studio e paziente, sull’importanza della duplice terapia antiaggregante, sull’importanza di non sospenderla senza indicazione del cardiologo curante, sulla possibilità che compaiano sanguinamenti “nuisance” ma che questi non hanno impatto sulla prognosi e non devono essere considerati preoccupanti;
  2. colloquio di almeno 15 minuti, tra medico dello studio e almeno un familiare stretto, sull’importanza della duplice terapia antiaggregante, sull’importanza di non sospenderla senza indicazione del cardiologo curante, sulla possibilità che compaiano sanguinamenti “nuisance” ma che questi non hanno impatto sulla prognosi e non devono essere considerati preoccupanti;
  3. una brochure informativa che descrive vantaggi ed effetti collaterali legati dalla duplice terapia antiaggregante, così come la loro gestione;
  4. una brochure indirizzata al Medico di Medicina Generale finalizzata alla presentazione di razionale e gestione della duplice terapia antiaggregante. Inoltre unno dei membri dello studio contatterà telefonicamente o via mail il Medico di Medicina Generale per spiegare il quadro clinico del paziente e le informazioni contenute nella brochure;
  5. un numero di telefono da contattare per poter discutere gli eventuali effetti collaterali della duplice terapia antiaggregante da contattare prima di qualunque decisione riguardante la sospensione della terapia stessa. Il numero di telefono sarà attivo dal lunedì al venerdì dalle 9 alle 12. Uno study coordinator riceverà le chiamate e, se necessario, contatterà uno dei medici per un colloquio telefonico con il paziente;
  6. due chiamate al mese da parte di uno study coordinator per controllare la compliance alla duplice terapia antiaggregante e I sanguinamenti BARC 1.

**PUNTI CARDINE DI DIFFERENZA TRA PRATICA STANDARD E PRATICA SPERIMENTALE.**

Le principali differenze che caratterizzano il braccio sperimentale sono:

- il medico di reparto sarà affiancato dal medico dello studio per rafforzare l’importanza che assume in un paziente trattato con angioplastica coronarica la terapia antiaggregante.
- L’intervento sarà più duraturo nel tempo, permettendo di soffermarsi meglio sui dettagli della terapia antiaggregante e dell’importanza di una adeguata compliance alla stessa
- L’intervento di comunicazione non sarà fatto solo al paziente ma anche a un familiare stretto che interagisce quotidianamente con il paziente
- Sarà messo a disposizione del materiale informativo che il paziente e i familiari potranno leggere insieme al medico dello studio e pertanto essere aiutati nella comprensione
- Nello specifico i 60 minuti di counselling saranno incentrati oltre che sui benefici potenziali della terapia antiaggregante, sui potenziali effetti collaterali. Questi solitamente non vengono mai discussi. Lo scopo dello studio è proprio quello di cercare di informare il paziente e i familiari su quali possano essere gli effetti collaterali, quali sono severi e quali no. Inoltre si spiegherà nel dettaglio come gestirli, quali sono segnali preoccupanti e quali sono gli aspetti che devono portare a interpellare il medico di medicina generale o il Pronto Soccorso.
- L’attività di counselling sarà principalmente dedicata alla descrizione dei sanguinamenti minori. Come distinguerli da quelli più importanti dal punto di vista prognostico e soprattutto si cercherà di far capire che la sospensione della terapia antiaggregante non li risolve o che se lo fa, è correndo un rischio significativo di reinfarto o morte improvvisa.

Durante la visita di follow-up a 1 mese verrà sottoposto a tutti i pazienti un questionario per indagare se si sono verificati sanguinamenti “nuisance” e determinare l’impatto che questi hanno sulla qualità di vita del paziente. Contemporaneamente sarà somministrato un questionario per valutare e stimare altri potenziali effetti avversi legati alla duplice terapia antiaggregante (es. dispnea da ticagrelor).

**Materiali e metodi**

I pazienti prima della dimissione dal reparto di Cardiologia saranno edotti sulla natura dello studio e, se concordi a partecipare allo studio, previa lettura del razionale e scheda informativa dello studio, firmeranno il consenso informato. Una copia della scheda informativa riguardante lo studio sarà lasciata al Paziente. I pazienti saranno arruolati in maniera consecutiva. La randomizzazione verrà eseguita mediante buste chiuse.

I pazienti randomizzati nel braccio sperimentale, in aggiunta alla normale routine clinica, riceveranno dal medico sperimentatore maggiori informazioni riguardanti la terapia antiaggregante e i sanguinamenti “nuisance”. Inoltre verrà fornito loro materiale informativo e didattico.

Nella pagina successiva e in allegato (Allegato 1) è riportata la scheda riassuntiva informativa che verrà rilasciata ai pazienti randomizzati a braccio sperimentale.

Allegato 1

**Riassunto schematico dello studio.**

Ricovero per cardiopatia ischemica

PCI con impianto di almeno 1 stent e indicazione ad almeno 6 mesi di DAPT

Consegna di lettera di dimissione (procedura standard)

Counselling e consegna di materiale informativo

(procedura sperimentale)

Follow-up 1 mese: valutazione cardiologica, questionario sui sanguinamenti e effetti avversi, QoL, registrazione di ogni potenziale evento avverso cardiologico e non

**OBIETTIVO PRIMARIO DELLO STUDIO.**

Follow-up 6 mesi: questionario sui sanguinamenti e effetti avversi, QoL, registrazione di ogni potenziale evento avverso cardiologico e non

- Stabilire se la procedura sperimentale migliora la qualità di vita a 1 mese, tramite questionario EQ-5D, in pazienti trattati con PCI con impianto di almeno 1 stent e in DAPT.

**OBIETTIVI SECONDARI DELLO STUDIO.**

- Stabilire se la procedura sperimentale migliora la qualità di vita, tramite questionario EQ-5D, a 6 mesi in pazienti trattati con PCI con impianto di almeno 1 stent e in DAPT.
- Stabilire se la procedura sperimentale riduce gli accessi al Pronto Soccorso e dal Medico di Medicina Generale.
- Stabilire se la procedura sperimentale riduce il numero di sospensioni (transitorie o permanenti) premature della DAPT.
- Valutare l’incidenza di sanguinamenti “nuisance” in differenti sottogruppi di popolazione (es. diabetici, BPCO, insufficienza renale cronica, sesso femminile)
- Valutare l’incidenza di effetti collaterali dovuti alla duplice terapia antiaggregante in differenti sottogruppi di popolazione (es. diabetici, BPCO, insufficienza renale cronica, sesso femminile) e in relazione al farmaco assunto (clopidogrel vs. ticagrelor vs. prasugrel)
- Valutare l’incidenza di dispnea non di origine cardiologica in differenti sottogruppi di popolazione (es. diabetici, BPCO, insufficienza renale cronica, sesso femminile) e in relazione al farmaco assunto (clopidogrel vs. ticagrelor vs. prasugrel)

**CRITERI DI INCLUSIONE**

Per poter essere incluso nello studio devono essere presenti tutti i seguenti criteri:

- - Firma del consenso informato.
  - Ammissione in ospedale per cardiopatia ischemica (vd definizione).
  - Intervento di angioplastica coronarica con impianto di almeno 1 stent.
  - Indicazione a DAPT per almeno 6 mesi.

**CRITERI DI ESCLUSIONE**

- - Terapia cronica con anti-coagulanti orali.
  - Vasta emorragia attiva entro gli ultimi 30 giorni.
  - Intervento pianificato, incluso innesto di bypass aortocoronarico (CABG) come intervento programmato (ibrido), entro 6 mesi dall’inclusione nello studio.

**DEFINIZIONE DI cardiopatia ischemica.**

Il termine “cardiopatia ischemica” raggruppa una serie di quadri clinici che hanno in comune lo sviluppo di ischemia miocardica, ossia di una sofferenza o un danno delle cellule miocardiche conseguente a un insufficiente apporto di ossigeno rispetto alle loro richieste metaboliche.

L’aterosclerosi coronarica è di gran lunga la causa più frequente di cardiopatia ischemica ma può essere causata anche da uno spasmo coronarico, da alterazioni del microcircolo coronarico o da cause extracoronariche.

La cardiopatia ischemica può manifestarsi come:

- - cardiopatia ischemica cronica che a sua volta può avere varie manifestazioni cliniche: angina cronica stabile; angina microvascolare; angina variante; cardiomiopatia dilatativa postischemica.
  - sindrome coronarica acuta che, a sua volta, può avere tre diverse presentazioni cliniche: infarto miocardico con sopraslivellamento persistente del tratto ST; infarto miocardico senza sopraslivellamento persistente del tratto ST; angina instabile.

**DEFINIZIONE E METODI DI VALUTAZIONE DELLA QUALITA’ DI VITA.**

L’EuroQoL è uno strumento standardizzato che consente di misurare lo stato di

salute degli intervistati e la loro qualità della vita sulla base del quale è possibile valutare

l’assistenza sanitaria prestata, una tecnica o una tecnologia. L’EuroQol è fondato su un questionario semplice e breve costituito da due sezioni distinte: la prima in cui compaiono cinque items che riguardano lo stato di salute corrente dell’intervistato ciascuno dei quali prevede la possibilità di scegliere un livello di gravità. Gli items sono: 1. Mobilità, 2. Cura di sé, 3. Attività usuali, 4. Dolore/disagio, 5. Ansia/depressione.

La seconda sezione include una scala visuoanalogica (VAS) rappresentata graficamente come un termometro graduato da 0 (stato di salute peggiore possibile) a 100 (stato si salute migliore possibile) e sulla quale l’intervistato indica il livello percepito del proprio stato di salute. [1]

Per questo studio abbiamo deciso di utilizzare una forma adattata del questionario EQ-5D già validata in letteratura (Allegato 2). La prima parte del questionario è stata ideata per mettere in evidenza la presenza di sanguinamenti “nuisance” e le azioni che sono state intraprese dal pazienti a fronte di questi sanguinamenti. La seconda parte è stata presa direttamente dal questionario EQ-5D e gli items presi in considerazione sono gli stessi: ansia/depressione, insoddisfazione/dolore, vestire/cure personali, hobbies/attività lavorativa/rapporti interpersonali/attività sessuale, mobilità. Come da studio precedente noi distingueremo 3 macro-aree: dolore/discomfort, ansia/depressione e VAS. Per le macro-aree dolore/discomfort e ansia/depressione, per ogni domanda è possibile scegliere un livello di gravità: no, moderatamente, estremamente. Per la macro-area VAS il paziente esprimerà il proprio stato di salute utilizzando un punteggio da 0 a 100.

Allegato 2

**DEFINIZIONE DI DISPNEA DA TICAGRELOR**

Il ticagrelor è un antiaggregante piastrinico con struttura simile a quella dell’adenosina, che agisce inibendo il recettore piastrinico P2Y12 dell’adenosina difosfato (ADP) con conseguente inibizione dell’aggregazione piastrinica.

Pertanto il suo meccanismo d’azione e la struttura chimica suggeriscono che il profilo di effetti avversi sia simile a quello dell’adenosina, che può causare dispnea (a volte con broncospasmo), rallentamento della conduzione atrio-ventricolare ed effetti vasodilatatori.

L'incidenza di dispnea nei pazienti trattati con ticagrelor è un evento piuttosto comune, generalmente lieve, transitorio e autolimitante. In genere insorge precocemente (entro la prima settimana di trattamento) e può essere parossistica, della durata di pochi minuti, oppure sostenuta per settimane. Nonostante ciò non sembra essere associata ad alcuna seria sequela sia polmonare che cardiaca.

Di seguito è riportato il questionario che utilizzeremo per indagare la dispnea da ticagrelor (Allegato 3)

**VARIABILI MONITORATE (si specifica che le variabili laboratoristiche rientrano nella normale routine clinica per la gestione di questi pazienti)**

|  | Dimissione | A 1 mese | A 6 mesi |
| --- | --- | --- | --- |
| Mini routine | x | x |  |
| ECG | x | x |  |
| Esame obiettivo | x | x |  |
| Anamnesi cardiovascolare | x |  |  |
| Terapia cardiologica in atto | x | x | x |
| Randomizzazione | x |  |  |
| Counselling e consegna del  materiale informativo | x |  |  |
| Questionario EQ-5D e VAS |  | x | x |
| Eventi Avversi | x | x | x |

**EVENTI CLINICI MONITORATI NEL FOLLOW-UP E RELATIVE DEFINIZIONI.**

- Morte
- Infarto miocardico
- Trombosi di stent
- Sanguinamenti definiti secondo la classificazione BARC
- Chirurgia minore e maggiore
- Interventi odontoiatrici
- Interventi oculistici
- Contatti non previsti con il Medico di Medicina Generale
- Accessi in Pronto Soccorso
- Sospensioni transitorie o permanenti di farmaci antiaggreganti

**DEFINIZIONE DI INFARTO MIOCARDICO ACUTO (LGESC 2012)**

Il termine infarto miocardico acuto dovrebbe essere impiegato in caso di evidenza di necrosi miocardica in un quadro clinico compatibile con ischemia miocardica acuta. E in particolare si deve verificare riscontro di movimento enzimatico tipico preferenzialmente delle troponina al di sopra del 99esimo percentile rispetto al limite di norma e in presenza di almeno una delle seguenti condizioni:

- - sintomi di ischemia,
  - modificazioni de novo del tratto ST e delle onde T, insorgenza di blocco di branca sinistro, sviluppo di onde Q patologiche al tracciato ECG.
  - evidenza di perdita di miocardio vitale all’imaging o riscontro di nuovi difetti della cinetica regionale,
  - identificazione di un trombo intracoronarico alla coronarografia.

**DEFINIZIONE DI TROMBOSI DI STENT**

In accordo con il documento di consenso e tutti i principali clinical trial randomizzati, la trombosi di stent sarà classificata come suggerito dall’ Academic Research Consortium (ARC). Di seguito è riportata la tabella con la classificazione dell’ARC della Trombosi di stent.


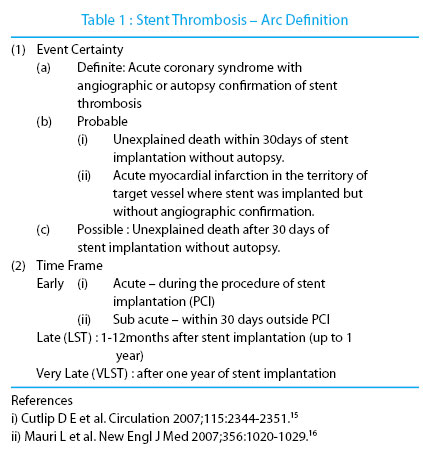


**DEFINIZIONE DI CLASSIFICAZIONE BARC**

Per le complicanze emorragiche sarà applicata la classificazione BARC (Bleeding Academic Research Consortium). Di seguito è riportata una tabella con la classificazione BARC.


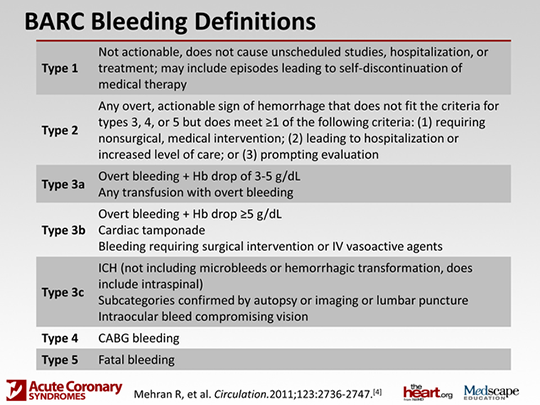


**SAMPLE SIZE**

Per il calcolo del sample size useremo come riferimento il lavoro di Amin et al. [2]

L’endpoint primario è calcolato sulla VAS.

Dai dati pubblicati [2] e da nostre valutazioni preliminari possiamo stimare che a un mese di distanza dalla procedura i pazienti in duplice terapia antiaggregante esprimano un valore medio di VAS di circa 75±19. Noi ipotizziamo che l’intervento di counselling incrementi dell’ 8% i valori di VAS. Pertanto sarà necessario arruolare almeno 211 pazienti per gruppo (α=0.05 e β=0.15, potenza statistica 90%). Il sample size è stato aumentato a 224 pazienti per gruppo per possibili drop-out, perdite al follow-up e ritiri del consenso.

**Piano statistico**

Tutte le analisi statistiche saranno svolte in modo indipendente dal Centro di Ricerca Clinica ed Epidemiologica dell’università degli Studi di Ferrara. Le variabili continue con distribuzione normale saranno espresse come media±deviazione standard. Le variabili continue a distribuzione non normale saranno espresse con la media e il range interquartile. La normalità della distribuzione sarà testata con il test di Kolmogorov–Smirnov o con la stima visuale del Q–Q Plot. Le variabili categoriche saranno espresse come numero e percentuale (%). Il confronto tra le variabili sarà eseguito con il χ2 test (Yates correction) se categoriche, con il test di Student o il test Satterthwaite o con l’ANOVA a una via se continue a distribuzione normale, con il test di Kruskal–Wallis se continue a distribuzione non normale. Attraverso il metodo di Kaplan-Meier saranno descritte le sopravvivenze libere da eventi avversi e le differenze tra gruppi saranno stimate e calcolare con il log-rank test. Le stime aggiustate per tutti i potenziali fattori di confondimento saranno ottenute con i Cox proportional hazard models, includendo tutte le variabili positive all’analisi uni variata o significative clinicamente. Un valore di p <0.05 sarà considerato significativo. Quando appropriato saranno calcolati gli intervalli di confidenza la 95% (95%CIs). Tutte le analisi saranno eseguite con STATISTICA 8 o STATA 10.

**REFERENZE BIBLIOGRAFICHE.**

1. R. Rabin, F. De Charro “EQ-5D: A Measure of Health Status from the EuroQol Group”. The Finnish Medical Society Duodecim, Ann. Med. 2001; 33:337-343.
2. Amit P. Amin, MD, MSC, Alok Bachuwar, MD, Kimberly J. Reid, MS, Adnan K. Chhatriwalla, MD, Adam C. Salisbury, MD, MSC, Robert W. Yeh, MD, MSC, Mikhail Kosiborod, MD, Tracy Y. Wang, MD, MHS, Karen P. Alexander, MD, Kensey Gosch, MS, David J. Cohen, MD, MSC, John A. Spertus, MD, MPH, Richard G. Bach, MD. Nuisance Bleeding With Prolonged Dual Antiplatelet Therapy After Acute Myocardial Infarction and its Impact on Health Status. JACC Vol. 61, No. 21, 2013 May 28, 2013:2130–8.
